# Supplementary material for: Enantioselective synthesis of a costic acid analogue with acaricidal activity against the bee parasite Varroa destructor
Source: R Soc Open Sci. 2020 Sep 9;7(9):200612. doi: 10.1098/rsos.200612 (PMC7540747; doi:10.1098/rsos.200612)
Supplement: Georgiladaki et al Electronic Supplementary Material RSOS 2020 [file rsos200612supp1.docx]

Enantioselective Synthesis of 2-[(2R,4aR,8aR)-4a-methyl decahydro-2-naphthalenyl]butanedioic acid as a Costic Acid Analog with Acaricidal Activity against the Bee Parasite *Varroa destructor*. †

S. Georgiladaki,^a^ D. Isaakidis, ^a^ A. Spyros, ^a^ G. K. Tsikalas^a^ and H. E. Katerinopoulos*^a^

^a^Department of Chemistry, University of Crete, Voutes Campus, 71003, Heraklion, Crete, Greece.

E-mail: kater@chemistry.uoc.gr; Fax: +30 2810 545166; Tel: +30 2810 545026

TABLE OF CONTENTS

| **General Procedures** | S2 |
| --- | --- |
| Preparation of compounds ***trans-*2 and *trans-*2a** | S3 |
| Preparation of compounds ***trans***-**4a,b** | S4 |
| Preparation of compounds ***trans***-**5a,b** | S5 |
| Preparation of compound ***trans***-**6** | S5 |
| Preparation of compound ***trans***-**7** | S6 |
| Preparation of compounds **10, 11** and **12** | S7 |
| Preparation of compound **4a*R*-1** | S8 |
| Preparation of compound (**4a*R*,8a*R)*-2** | S9 |
| Preparation of compound (**4a*R*,8a*R)*-4a,b** | S10 |
| Preparation of compound (**4a*R*,8a*R)*-5a,b** | S11 |
| Preparation of compound **(2*R*,4α*R*,8α*R*)-6** | S12 |
| Preparation of compound **(2*R*,4α*R*,8α*R*)-7** | S13 |
| NMR SPECTRA | S14 |
| Chiral GC and Chiral HPLC analysis of enantiomers | S33 |
| References | S36 |

**General Procedures:** General procedures that were followed have been published elsewhere [1]. All reactions were carried out under anhydrous conditions in dry solvents, using argon or nitrogen in flame-dried glassware. Reactions were monitored by thin-layer chromatography (TLC) using silica gel plates from Merck (60F254), which were visualized under a UV-Vis Lamp (254 and 366 nm, respectively) or with a 7% ethanolic solution of phosphomolybdic acid. Flash column chromatography was performed in silica gel 60 from Merck (230-400 mesh). NMR spectra were taken on an AMX500 Bruker FT-NMR or a MSL300 Bruker FT-NMR spectrometer; proton chemical shifts are reported in relative to tetramethylsilane. All mass spectra were acquired using sonic-spray ionization (SSI) mass spectrometry (MS). Absolute mass spectra were taken on a LTQ ORBITRAP XL with ETD- Thermo Fisher Scientific (San Jose,CA,USA-Bremen,Germany) with son source: Electrospray Ionization (ESI) positive mode and an Orbitrap Mass Analyser. The enantiomeric excess of the compounds was determined by HPLC using DAICEL columns with chiral static phase (Chiralpak AS-H) using as mobile phase hexane and a detection wavelength of 240 nm. GC analysis on a chiral column was also performed using a GC-2014 SHIMADZU instrument with a JW Chiral CP-Chrasil Dex CB column, a hellion flow of 1.40mL/min at a temperature of 150 ^ο^C. Optical rotation values were measured on the P3000 (A. Krüss Optronic) polarimeter. Specific rotation values, [α]_D_^T^, refer to a concentration of g/100mL and the polarimeter cell length was 50 mm. The symbol D refers to the D-line of Sodium (589 nm) and the temperature (T) is given in degrees Celsius (^ο^C).

**Synthetic Procedures**

The synthesis of compounds **1-7** is not described in this section since the preparation of **1** is known (reference 2 and the literature cited therein), and the synthetic procedures of compounds **2-7** are similar to those described in the following experimental section.

**7:** ^1^H-NMR (500MHz, CDCl_3_*))*: δ= 11.21 (s, 1H), 10.33 (s, 1H), 2.80-2.71 (m, 2H), 2.56-2.48 (m, 1H), 1.75-1.66 (m, 2H), 1.52-1.43 (m, 3H), 1.41-1.32 (m, 3H), 1.26-1.20 (m, 3H), 1.11 (dq, J1=23.2 Hz, J2=11Hz, 3H), 1.06-0.98 (m, 2H), 0.78 (s, 3H)

^13^C-ΝΜR (500MHz, CDCl_3_): δ= 181.04/181.00 (two diastereoisomers), 179.1, 46.8, 45.2/45.1 (two diastereoisomers), 41.4, 40.3/40.2 (two diastereoisomers), 33.6, 33.2/33.1 (two diastereomers), 32.1, 28.90/28.88 (two diastereoisomers), 26.9, 26.01, 25.2, 21.9, 15.8

MS: m/z: 268 (M+). Exact mass; [M+H]^+^ Calcd: 269.1747, observed: 269.1742.

**Preparation of compounds** ***trans-*2 and *trans-*2a**: To a flame dried three-necked 500mL flask, were added 50ml of dry diethyl ether and 200 mg of compound (*4aR/S)*-**1** (1.22mmol). Ammonia gas was added to the system and liquefied at -78^o^C (2-propanol /dry ice) under vigorous stirring, for 15 minutes. The excess of ammonia was restrained by a silicon oil trap. Then 55mg Li were added portionwise under argon gas. Gradually the solution acquired a blue color, characteristic of the reaction. After 15 minutes stirring 368mg NH_4_Cl were gradually added. When the solution was completely decolorized, 50ml H_2_O were added and the mixture was extracted with ether and 5% aqueous HCl solution. The resulting mixture contained the desired ketone *trans*-**2** and the corresponding alcohol *trans*-**2a**. 200mg of this mixture were dissolved in 1,5ml of acetone. Then, 253μl of 8N Jones reagent were added, at ice bath temperature. The solution was stirred at 0^o^C for 3 hours, till the Jones oxidation was complete. The excess of Jones reagent was quenched by the addition of isopropyl alcohol and the mixture was extracted with ether and H_2_O. The product was isolated from the crude mixture by flash chromatography on silica gel using ethyl acetate: petroleum ether = 1: 9 as eluent. The reaction yield was 176 mg (87%). Diastereomeric excess was found by GC analysis to be 92.8 %. Chiral GC analysis of the major diastereomer indicated the presence of a racemic mixture.

***trans-*2:** ^1^H-NMR (500MHz, CDCl_3_): δ= 2.39 (dt, *J*_1_=15Hz, *J*_2_=6.7Hz, 1H), 2.23 (qt, *J*_1_=20.5Hz, *J*_2_=15.5Hz, *J*_3_=5Hz, *J*_4_=2Hz, 1H), 2.11 (t, *J*=14.6Hz, 1H), 2.03 (dq, *J*_1_=4.3Hz, *J*_2_=2.1Hz, 1H), 1.70-1.65 (m, 1H), 1.62 (dq, *J*_1_=6.7Hz, *J*_2_=2Hz, 1H), 1.51-1.43 (m, 4H), 1.39 (dd, *J*_1_=13.6Hz, *J*_2_=5.1Hz, 1H), 1.31-1.26 (m, 1H), 1.22 (dd, *J*_1_=12.7Hz, *J*_2_=3Hz, 1H), 1.17 (dd, *J*_1_=10.6Hz, *J*_2_=3Hz, 1H), 1.04 (dt, *J*_1_=5Hz, *J*_2_=13.9Hz, 1H), 0.98 (s, 3H). ^13^C-ΝΜR (500MHz, CDCl_3_): δ= 211.6, 45.0, 44.7, 41.1, 40.4, 38.3, 33.1, 29.0, 26.0, 21.6, 14.9

Exact mass; [M+H]+ Calcd: 167.1436, observed: 167.1433.

***trans-*2a:** MS: m/z: 168 (M^+^)

**Preparation of compounds** ***trans***-**4a,b:** To a 25ml round bottom flask were transferred ketone *trans*-**2** (100mg, 0.60mmol) and dimethyl succinate (130mg, 0.90mmol). Benzene (3ml) was added and the mixture was distilled in a rotary evaporator, and then vacuum pumped for 1 hour, to remove traces of moisture.

In a flame dried 25mL round bottom flask equipped with condenser were added 5ml of dry *tert*-butanol and the system temperature was increased to 30 °C. Then Na (30mg) was added in small portions, and the system was heated to 90 ^o^C until the metal dissolved. The two solutions were transferred to an autoclave and were stirred at 100 - 110 °C for 20 h. The solution was then neutralized by the addition of 6N HCl (2ml) and extracted with dichloromethane (3x20ml). The organic layer was washed with saturated NaCl solution (3x10ml), dried with anhydrous MgSO_4_ and the solvent was removed *in vacuo*. The products were isolated from the crude mixture by flash chromatography on silica gel using ethyl acetate: petroleum ether = 1: 4 as eluent. The reaction yield was 134 mg (70%).

***trans***-**4a,b:** ^1^H-NMR (500MHz, CDCl_3_): δ= 9.98 (br.s, 1H), 5.51-5.46 (m, 1H), 3.27 (dq, *J*_1_=9.6Hz, *J*_2_=5.9Hz, 1H), 2.88 (tq, *J*_1_=9.6Hz, *J*_2_=4.5Hz, 1H), 2.44 (dd, *J*_1_=16.7Hz, *J*_2_=5.7Hz, 1H), 1.80 (dq, *J*_1_=17.6Hz, *J*_2_=4.5Hz, 3H), 1.70 (t, *J*_1_=*J*_2_=14.4Hz, 2H), 1.55-1.44 (m, 3H), 1.404/1.400 (s, 9H for all diastereoisomers), 1.33 (dt, *J*_1_=11Hz, *J*_2_=4.3Hz, 1H), 1.23-1.16 (m, 2H), 1.61-1.01 (m, 2H), 0.736/0.728 (s, 3H for all diastereoisomers)

^13^C-ΝΜR (500MHz, CDCl_3_): δ= 178.5, 172.2/172.1(two diastereoisomers), 133.4/133.3 (two diastereoisomers), 124.5/124.0 (two diastereoisomers), 80.93/80.90 (two diastereoisomers), 49.3/49.2 (two diastereoisomers), 42.3, 41.06/41.03 (two diastereoisomers), 40.4/40.3 (two diastereoisomers), 35.4/35.1 (two diastereoisomers), 32.2, 32.1/32.0 (two diastereoisomers), 31.0, 29.0/28.9 (two diastereoisomers), 28.04/28.00 (two diastereoisomers), 27.99, 26.8, 22.3, 16.16/16.08 (two diastereoisomers).

**Preparation of compounds** ***trans***-**5a,b:** To a flame dried two-necked 25mL flask equipped with condenser, were added *trans*-**4a,b** (90mg, 0.28mmol) dissolved in 1ml dry methanol. Then the system was cooled to 0^o^C and concentrated H_2_SO_4_ (0.009ml, 0.13mmol) was added. The solution was stirred at 0 ^o^C for 30 minutes and the temperature was gradually increased to room temperature. Then, the solution was heated at 70^o^C for 2 hours. The solution was extracted with diethyl ether (3x20ml) and the organic layer was washed with saturated NaCl solution (3x10ml), dried with anhydrous MgSO_4_ and the solvent was removed *in vacuo*. The product was isolated from the crude mixture by flash chromatography using 25% ethyl acetate (EA) in petroleum ether (P.E.) as eluent. The reaction yield was 61 mg (65%).

^1^H-NMR (500MHz, CDCl_3_): δ= 5.54-5.45 (m, 1H), 3.64 (s, 3H), 3.30 (dq, *J*_1_=9.2Hz, *J*_2_=5.9Hz, 1H), 2.83 (dq, *J*_1_=9.3Hz, *J*_2_=6.5Hz, 1H), 2.42 (dq, *J*_1_=6.3Hz, *J*_2_=2.8Hz, 1H), 1.80 (dq, *J*_1_=18.1Hz, *J*_2_=4.4Hz, 2H), 1.75-1.62 (m, 3H), 1.54-1.44 (m, 2H), 1.410/1.406 (s, 9H for all diastereoisomers), 1.36-1.30 (m, 1H), 1.21 (qt, *J*_1_=32.8Hz, *J*_2_=20.8Hz, *J*_3_=7.4Hz, *J*_4_=4.4Hz, 2H), 1.15-1.09 (m, 2H), 0.94-0.81 (m, 1H), 0.738/0.729 (s, 3H for all diastereoisomers)

^13^C-ΝΜR (500MHz, CDCl_3_): δ= 172.74/172.72 (two diastereoisomers), 172.3/172.2 (two diastereoisomers), 133.6/133.5 (two diastereoisomers), 124.3, 123.8, 80.7, 51.7, 49.55/49.51 (two diastereoisomers), 42.4, 41.08/41.05 (two diastereoisomers), 40.4/40.3 (two diastereoisomers), 35.4, 35.1, 32.1/32.03 (two diastereoisomers), 31.0, 28.96/28.94 (two diastereoisomers), 28.10/28.06 (two diastereoisomers), 26.8, 22.3, 16.14/16.10(two diastereoisomers).

**Preparation of compound** ***trans***-**6:** To a flame dried two-necked 25mL flask were added *trans*-**5a,b** (90mg, 0.26mmol) dissolved in 9ml dry ethanol and PtO_2_ (30mg). The solution was stirred for 1 h under the pressure of gaseous H_2_. The catalyst was deactivated by addition of dichloromethane and was removed from the solution by filtration on celite. The solvent was removed *in vacuo* to give pure *trans*-**6**. The reaction yield was 86mg (98%). GC analysis of ***trans*-6** on a non-chiral column, indicated the presence of the two major diastereomers with a de of 67.8. HPLC analysis on chiral column, indicated the presence of two enantiomeric pairs, both as racemic mixtures.

***trans***-**6:** ^1^H-NMR (500MHz, CDCl_3_): δ= 3.64 (s, 3H), 2.66 (dq, *J*_1_=15.8Hz, *J*_2_=7Hz, 1H), 2.59 (tq, *J*_1_=10.9Hz, *J*_2_=3.5Hz, 1H), 2.38 (dq, *J*_1_=7.8Hz, *J*_2_=3.5Hz, 1H), 1.75-1.69 (m, 1H), 1.68-1.57 (m, 2H), 1.47-1.44 (m, 2H), 1.43(s, 9H), 1.38-1.36 (m, 1H), 1.35-1.33 (m, 1H), 1.31 (t, *J*_1_=*J*_2_=4.4Hz, 1H), 1.25-1.14 (m, 4H), 1.12-1.08 (m, 1H), 1.05 (dt, *J*_1_=10Hz, *J*_2_=5.2Hz, 2H), 1.01 (dt, *J*_1_=11.9Hz, *J*_2_=6.8Hz, 1H), 0.759 (s, 3H)

^13^C-ΝΜR (500MHz, CDCl_3_): δ= 173.64/173.60 (two diastereoisomers), 173.2, 80.6, 51.7, 47.9/47.8 (two diastereoisomers), 45.3/45.2 (two diastereoisomers), 41.7, 41.6, 41.55/41.53 (two diastereoisomers), 40.8/40.7 (two diastereoisomers), 33.8, 33.7, 33.6, 32.5, 29.02/29.0 (two diastereoisomers), 28.2, 27.0, 25.6/25.5 (two diastereoisomers), 21.9, 15.7

Exact mass; [M-C_4_H_9_]+ Calcd: 281.1752, observed: 281.1749.

**Preparation of compound** ***trans***-**7:** To a flame dried two-necked 25mL flask were added *trans*-**6** (50mg, 0.17mmol) dissolved in 2mL of dry DMSO and *t*-BuOK (80mg, 0.68mmol). The reaction was completed in 2 hours. Addition of diethyl ether precipitated the product in its potassium salt form. The solid was isolated by centrifugation, dissolved in dry acetonitrile, transferred to a flame dried flask and protonated using Amberlyst 15. The solvent was removed *in vacuo*. The reaction yield was 45mg (98%).

***trans***-**7:** ^1^H-NMR (500MHz, CDCl_3_): δ= 11.85 (s, 1H), 10.47 (s, 1H), 2.79-2.71 (m, 2H), 2.52 (dq, *J*_1_=9.3Hz, *J*_2_=3.5Hz, 1H), 1.78-1.66 (m, 2H), 1.53-1.44 (m, 3H), 1.38 (dq, *J*_1_=12.6Hz, *J*_2_=3.6Hz, 3H), 1.28-1.19 (m, 3H), 1.18-0.98 (m, 5H), 0.78 (br.s, 3H)

^13^C-ΝΜR (500MHz, CDCl_3_): δ= 181.25/181.21 (two diastereoisomers), 179.32/179.31 (two diastereoisomers), 46.9, 45.07/45.05 (two diastereoisomers), 41.35/41.32 (two diastereoisomers), 40.13/40.10 (two diastereoisomers), 33.5, 33.3/32.9 (two diastereoisomers), 32.0, 28.81/28.77 (two diastereoisomers), 26.9, 25.9, 25.1, 21.8, 15.6

Exact mass; [M+H]^+^ Calcd: 269.1752, observed: 269.1749.

**Preparation of compound** **10:** A Dean-Stark water separator and condenser were applied to a 250mL round bottom flask containing magnetic stirrer. 2-methylcyclohexanone ± **8** (20.0mL, 164.9 mmol) and (S)-(-)-α-methylbenzylamine (**9)** (21.2mL, 164.9 mmol ) were added dissolved in 100mL of dry toluene. The flask was placed in an oil bath and heated to 125 °C under reflux conditions and argon gas for 24 hours and kept overnight in agitation. A GC-MS spectrum revealed that the desired intermediate was formed at 93%. The solvent was removed *in vacuo.* The crude product was used directly in the next reaction.

MS: m/z: 215 (M^+^)

**Preparation of compound** **11:** To a flame dried round-bottomed flask 250mL flask, was added compound **10** (28.40 g, 131.92mmol) dissolved in 75mL of THF. The flask was placed in an ice bath and 3-butene-2-one was added (11.55mL, 138.50mmol) dropwise and under vigorous stirring. After the addition was complete, the ice bath was maintained for 30 minutes and then the solution remained stirred for 5 days at 20 °C. This reaction required special conditions because of the convenience of mvk producing polymerization products. Finally, the solution was extracted with diethyl ether (3x20ml) and the organic layer was washed with saturated NaCl solution (3x10ml) and dried with anhydrous MgSO_4_ and the solvent was removed *in vacuo*. The crude product was used directly in the next reaction.

MS: m/z: 285 (M^+^)

**Preparation of compound** **12:** To a 250mL round-bottomed flask equipped with a magnetic stirring bar and placed in an ice bath, compound **11** was added (38.0 g) with 4mL of acetic acid, which had previously been diluted in 16mL of deionized H_2_O (20% solution). The solution was kept in an ice bath for 15 minutes and then, vigorously stirred for 2 hours. The solution was extracted with CH_2_Cl_2_ (3x20ml) and the organic layer was washed with saturated NaCl solution (3x10ml) and dried with anhydrous MgSO_4_ and the solvent was removed *in vacuo*. It is worth mentioning that the GC-MS mass spectrum indicated as major product the bicyclic unsaturated ketone ***4aR-*1**- which was expected to form in a subsequent reaction via Robinson's cyclization - as it is possible that the hydrolyzed amine acted as a base to form the compound.

MS **54**: m/z: 182(M^+^)

**Preparation of compound** **4a*R*-1:** To a flame dried 25mL round bottom flask, 5mL of dry methanol and 320 mg of Na cut into small pieces were transferred. The addition of Na was at 0 °C while gradually the solution formed came to room temperature. To a second pre-dried 250mL round bottom flask was transferred compound **12** (12g, 65.90 mmol) dissolved in 70mL of dry methanol. The temperature of this solution was lowered to 0 °C and the sodium methoxide solution, which was also at 0 ° C, was gradually added. The solution was allowed to gradually come to 25 ° C and then heated to 70 °C for 20 hours. The solution was neutralized by the addition of acetic acid to pH = 7 and was extracted with CH_2_Cl_2_ (3x20ml) and the organic layer was washed with saturated NaCl solution (3x10ml) and dried with anhydrous MgSO_4_ and the solvent was removed *in vacuo*. The product was isolated from the crude mixture by flash chromatography on silica gel using ether: petroleum ether = 1: 4 as eluent. The desired **4a*R* -1** product was formed in 37% (4.09g). The optical rotation of the substance was determined as [α] ^20^_D_ = - 238° (EtOH, c = 1.0) corresponding to 99% ee according to the literature data.

^1^H-NMR (500MHz, CDCl_3_): δ= 5.69 (s, 1H), 2.47 (dq, *J*_1_=14.2Hz, *J*_2_=5.5Hz, 1H), 2.37-2.28 (m, 2H), 2.22 (dt, *J*_1_=14.6Hz, *J*_2_=2Hz, 1H), 1.90-1.82 (m, 1H), 1.79 (dd, *J*_1_=14.2Hz, *J*_2_=4.6Hz, 1H), 1.74 (dq, *J*_1_=5.6Hz, *J*_2_=3.1Hz, 1H), 1.64 (dt, *J*_1_=7.4Hz, *J*_2_=3Hz, 3H), 1.40-1.28 (m, 2H), 1.209 (s, 3H)

^13^C-ΝΜR (500MHz, CDCl_3_): δ= 198.9, 170.0, 123.7, 41.2, 37.7, 35.5, 33.6, 32.4, 26.8, 21.7, 21.4.

MS: m/z: 164 (M^+^)

This data is in full agreement with the bibliographic data.

**Preparation of compound (4a*R*,8a*R)*-2**

To a 500mL three-necked round bottomed flask, were transferred 600 mg, (3.65 mmol) of compound (**4a*R*)-1** in 50mL of diethyl ether. Maintaining vigorous stirring, ammonia gas was added to the solution, and was liquefied and mixed with the ether solution by means of a dry ice - 2-propanol bath which kept the temperature of the system at -78 °C. Ammonia gas was introduced into the solution for about 15 ' while its excess was collected by a silicone oil trap. The liquid ammonia flow was then discontinued; an inert gas supply was connected to the flask, and the addition of 170 mg of Li, was added in portions. Gradually the solution became blue, characteristic of the specific reaction (solvated electron). After 15 min 368 mg of NH_4_Cl were added portion wise. When the solution was completely decolorized, 50mL of H_2_O were added, and the system was extracted with ether and 5% aqueous HCl. The resulting mixture contained the desired ketone (**4a*R*,8a*R)*-2** as well as the corresponding alcohol. The whole mixture (200 mg) was subjected to Jones oxidation: it was dissolved in 1.5mL of acetone, cooled in an ice bath, and then 1,0mL of 8N Jones reagent was added to the acetone solution. The system temperature was maintained at 0 °C for 3 hours, whereupon the reaction was complete. Excess Jones reagent was neutralized by the addition of isopropyl alcohol and the mixture was extracted with ether and H_2_O. Separation of the mixture was accomplished by column chromatography, on silica gel, using as eluent ethyl acetate: petroleum ether = 1: 9. The yield of the reaction was 79% (480mg)_._ The optical rotation of the new product was determined as [α]^20^_D_= - 42,0° (EtOH, c=1,0). GC analysis of **(*4aR,8aR*)-2** using chiral column indicated an ee of 90.8%.

(**4a*R*,8a*R)*-2:** ^1^H-NMR (500MHz, CDCl_3_): δ= 2.38 (dt, *J*_1_=15Hz, *J*_2_=6.7Hz, 1H), 2.22 (qt, *J*_1_=20.5Hz, *J*_2_=15.5Hz, *J*_3_=5Hz, *J*_4_=2Hz, 1H), 2.10 (t, *J*_1_= *J*_2_=14.6Hz, 1H), 2.01 (dq, *J*_1_=4.3Hz, *J*_2_=2.1Hz, 1H), 1.69-1.64 (m, 1H), 1.61 (dq, *J*_1_=6.7Hz, *J*_2_=2Hz, 1H), 1.52-1.43 (m, 4H), 1.39 (dd, *J*_1_=13.6Hz, *J*_2_=5.1Hz, 1H), 1.31-1.27 (m, 1H), 1.21 (dd, *J*_1_=12.7Hz, *J*_2_=3Hz, 1H), 1.17 (dd, *J*_1_=10Hz, *J*_2_=3Hz, 1H), 1.03 (dt, *J*_1_=5Hz, *J*_2_=13.9Hz, 1H), 0.98 (s, 3H)

^13^C-ΝΜR (500MHz, CDCl_3_): δ= 211.0, 44.7, 44.4, 40.8, 40.1, 38.0, 32.9, 28.8, 25.8, 21.4, 14.7

Exact mass; [M+H]^+^ Calcd: 167.1436, observed: 167.1429.

Preparation of compound (**4a*R*,8a*R)*-4a,b**

In a 25mL round bottom flask was transferred (**4a*R*,8a*R)*-2 (**280 mg, 1.70 mmol**)** and dimethyl succinate (380.0 mg, 2.60 mmol). An amount of 2-3mL of benzene was added thereto, and the mixture was distilled off on a rotary evaporator and then left in the vacuum pump for about 1 hour to remove any solvent or moisture.

To another flame-dried 25mL round bottom flask equipped with a condenser were transferred 10mL of dry *tert*-butanol and the temperature was raised to 30 °C (to avoid solidification of *tert*-butanol (mp 25 to 26 °C) .This flask was then added Sodium (840 mg) cut into small pieces and the system was heated to 90 °C until the metal was dissolved.

Then, the above two solutions were transferred to an autoclave and heated by means of an oil bath to 100-110 °C for 20 hours. The solution was then neutralized by the addition of 2ml of 6N HCl and extracted with dichloromethane (3x20ml) and the organic layer was washed with saturated NaCl solution (3x10ml). The organic layer was dried with anhydrous MgSO_4_ and the solvent was removed using a rotary evaporator. The mixture was separated by column chromatography on silica gel eluting with ethyl acetate: petroleum ether = 1:4. It should be noted that the main the reaction product was the methyl ester. The desired products were formed in 70% (357mg).

(**4a*R*,8a*R)*-4a,b**: ^1^H-NMR (500MHz, CDCl_3_): δ= 10.70 (s, 1H), 5.52-5.45 (m, 1H), 3.644/3.643 (s, 3H for all diastereoisomers), 3.36 (dd, *J*_1_=14.7Hz, *J*_2_=8.1Hz, 1H), 2.92 (dd, *J*_1_=16.8Hz, *J*_2_=9.6Hz, 1H), 2.57 (t, *J*_1_=*J*_2_=6.7Hz, 1H), 2.48 (dq, *J*_1_=12.5Hz, *J*_2_=6.6Hz, 2H), 1.84-1.70 (m, 3H), 1.69-1.59 (m, 2H), 1.48-1.42 (m, 2H), 1.37-1.29 (m, 2H), 1.22-1.16 (m, 1H), 1.10-1.03 (m, 1H), 0.69/0.67 (s, 3H)

^13^C-ΝΜR (500MHz, CDCl_3_): δ= 178.6, 178.0, 173.5/173.4 (two diastereoisomers)*,* 171.5, 132.8/132.7 (two diastereoisomers)*,* 124.8/124.7 (two diastereoisomers)*,* 52.2/52.1 (two diastereoisomers)*,* 48.4, 48.0, 42.2, 41.0/40.9 (two diastereoisomers)*,* 40.2/40.1 (two diastereoisomers)*,* 35.4, 35.0, 31.93/31.87, 31.4/31.2, 30.1, 29.2, 28.7, 28.0, 26.7, 22.2, 16.1/16.0 (two diastereoisomers).

Preparation of compound (**4a*R*,8a*R)*-5a,b**

To a flame-dried, 25mL two neck round bottom flask equipped with a condenser were transferred 90 mg (0.28mmol) of the reactant dissolved in 1mL of dry methanol. The system was then cooled to 0 °C and then 9.0 μL concentrated H_2_SO_4_ was added in one portion. The solution remained for 30 minutes under stirring and gradually returned to ambient temperature. The solution was then heated under stirring to 70 °C for 2 hours. The solution was extracted with diethyl ether (3x20mL) and the organic layer was washed with saturated NaCl solution (3x10mL). The organic layer was dried with anhydrous MgSO_4_ and the solvent was removed in a rotary evaporator. The mixture was separated by column chromatography on silica gel eluting with ethyl acetate: petroleum ether = 1: 4. It is worth noting that the only product was the dimethyl ester (**4a*R*,8a*R)*-5a,b** (65%, 54mg).

(**4a*R*,8a*R)*-5a,b** : ^1^H-NMR (500MHz, CDCl_3_): δ= 5.53-5.47 (m, 1H), 3.665/3.663 (s, 3H for all diastereoisomers), 3.637/3.634 (s, 3H for all diastereoisomers), 3.41 (dt, *J*_1_=5.9Hz, *J*_2_=9.1Hz, 1H), 2.88(dq, *J*_1_=9.6Hz, *J*_2_=3.4Hz, 1H), 2.47 (dq, *J*_1_=13.4Hz, *J*_2_=5.9Hz, 1H), 1.88-1.77 (t, *J*=18Hz, 1H), 1.77-1.64 (m, 3H), 1.53-1.37 (m, 4H), 1.36-1.30 (m, 1H), 1.26-1.14 (m, 2H), 1.13-1.00 (m, 2H), 0.710/0.691 (s, 3H for all diastereoisomers).

Preparation of compound **(2*R*,4α*R*,8α*R*)-6**

In a flame-dried 25mL two-necked round-bottomed flask, were transferred 90mg (0.31mmol) of the mixture (**4a*R*,8a*R)*-5a,b** dissolved in 9.0mL of dry ethanol, and 30 mg of the catalyst (PtO_2_). The solution remained for 1 h, stirring under H_2_ atmosphere. The catalyst was inactivated by the addition of dichloromethane and removed from the solution by filtration through celite. The solvent was removed using a rotary evaporator. The yield of the reaction was 98% (89mg). GC analysis of **(2*R*,4α*R*,8α*R*)-6** on a non-chiral column, indicated the presence of two diastereomers with a de of 91.9 %. HPLC analysis on chiral column, indicated the presence of one enantiomer with retention time of 22.15 min, the same as the *first* of the two enantiomers of the major diastereomer in ***trans-*6.**

**(2*R*,4α*R*,8α*R*)-6:** ^1^H-NMR (500MHz, CDCl_3_): δ= 3.660 (s, 3H), 3.627 (s, 3H), 2.74-2.66 (m, 2H), 2.44 (dq, *J*_1_=8.7Hz, *J*_2_=4.2Hz, 1H), 1.69-1.60 (m, 2H), 1.47-1.39 (m, 3H), 1.38-1.27 (m, 4H), 1.20-1.11 (m, 3H), 1.10-1.03 (m, 2H), 1.02-0.95 (m, 2H), 0.739 (s, 3H)

^13^C-ΝΜR (500MHz, CDCl_3_): δ= 175.12, 175.08, 173.04, 173.02, 51.8, 51.69, 51.68, 46.98, 46.97, 45.1, 41.5, 41.4, 40.6, 40.5, 33.56, 33.54, 33.4, 32.8, 32.4, 28.92, 28.90, 26.9, 25.8, 25.5, 21.9, 15.70, 15.69, (one diastereoisomer).

MS: m/z: 296 (M^+^)^+^). Exact mass; [M+H]^+^ Calcd: 297.2060, observed: 297.2062; [M+Na]^+^ Calcd: 319.1874, observed: 319.1882.

Preparation of compound **(2*R*,4α*R*,8α*R*)-7**

To a pre-dried, 25mL two neck round bottom flask were transferred **(2*R*,4α*R*,8α*R*)-6 (**50 mg, 0.17mmol) dissolved in 2.0mL of dry dimethylsulfoxide and 80mg (0.68mmol) of potassium *tert*-butoxide. In 2 h the reaction mixture contained the final product as well as and the corresponding monoacids, whereupon it was allowed to stir for 10 additional hours to complete the reaction. Then, addition of diethyl ether precipitated the product in the di-potassium salt form. Centrifugation of this solution removed the DMSO from the salt, which was then dissolved in dry acetonitrile, transferred to a pre-dried flask and protonated using Amberlyst 15. The solvent was removed *in vacuo*. The yield of the reaction was 98% (45mg).

**(2*R*,4α*R*,8α*R*)-7:** ^1^H-NMR (500MHz, CDCl_3_): δ= 11.48 (br.s, 1H), 10.563 (br.s, 1H), 2.79-2.71 (m, 2H), 2.52 (dq, *J*_1_=9.2Hz, *J*_2_=4.2Hz, 1H), 1.78-1.66 (m, 2H), 1.52-1.44 (m, 3H), 1.38 (dd, *J*_1_=23.4Hz, *J*_2_=12Hz, 3H), 1.28-1.20 (m, 3H), 1.18-1.08 (m, 3H), 1.07-0.99 (m, 2H), 0.782 (s, 3H)

^13^C-ΝΜR (500MHz, CDCl_3_): δ= 181.2, 181.1, 179.2, 46.9, 45.20, 45.17, 41.5, 41.4, 40.3, 40.2, 33.6, 33.34, 33.27, 33.0, 32.1, 28.93, 28.90, 27.0, 26.1, 25.2, 21.9, 15.7.

MS: m/z: 268 (M+). Exact mass; [M+H]^+^ Calcd: 269.1747, observed: 269.1748; [M+Na]^+^ Calcd: 291.1561, observed: 291.1566.

**^1^H NMR spectrum of compound *trans* 2 (500 MHz, CDCl_3_)**


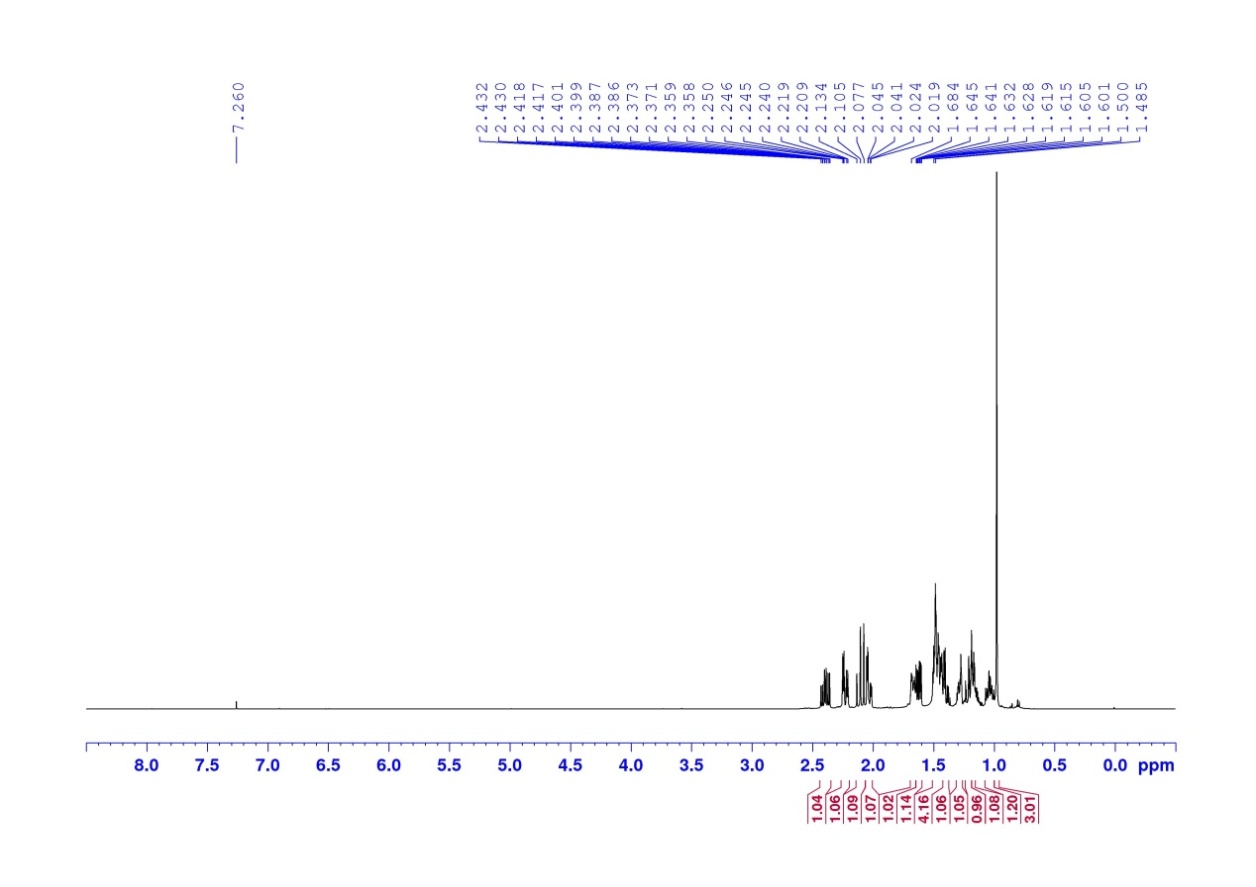


**^13^C NMR spectrum of compound *trans* 2 (500 MHz, CDCl_3_)**


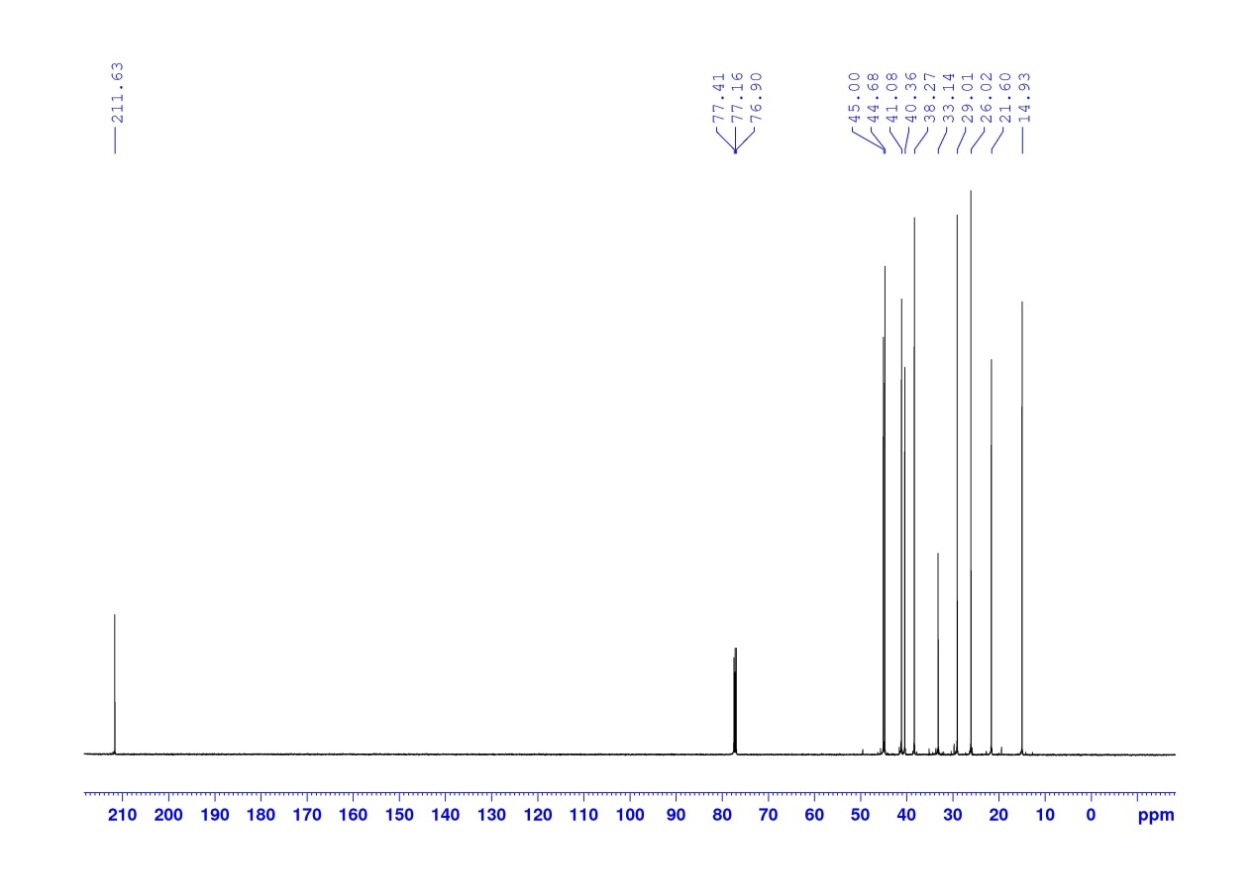


**^1^H NMR spectrum of compound *trans* 4a,b (500 MHz, CDCl_3_)**


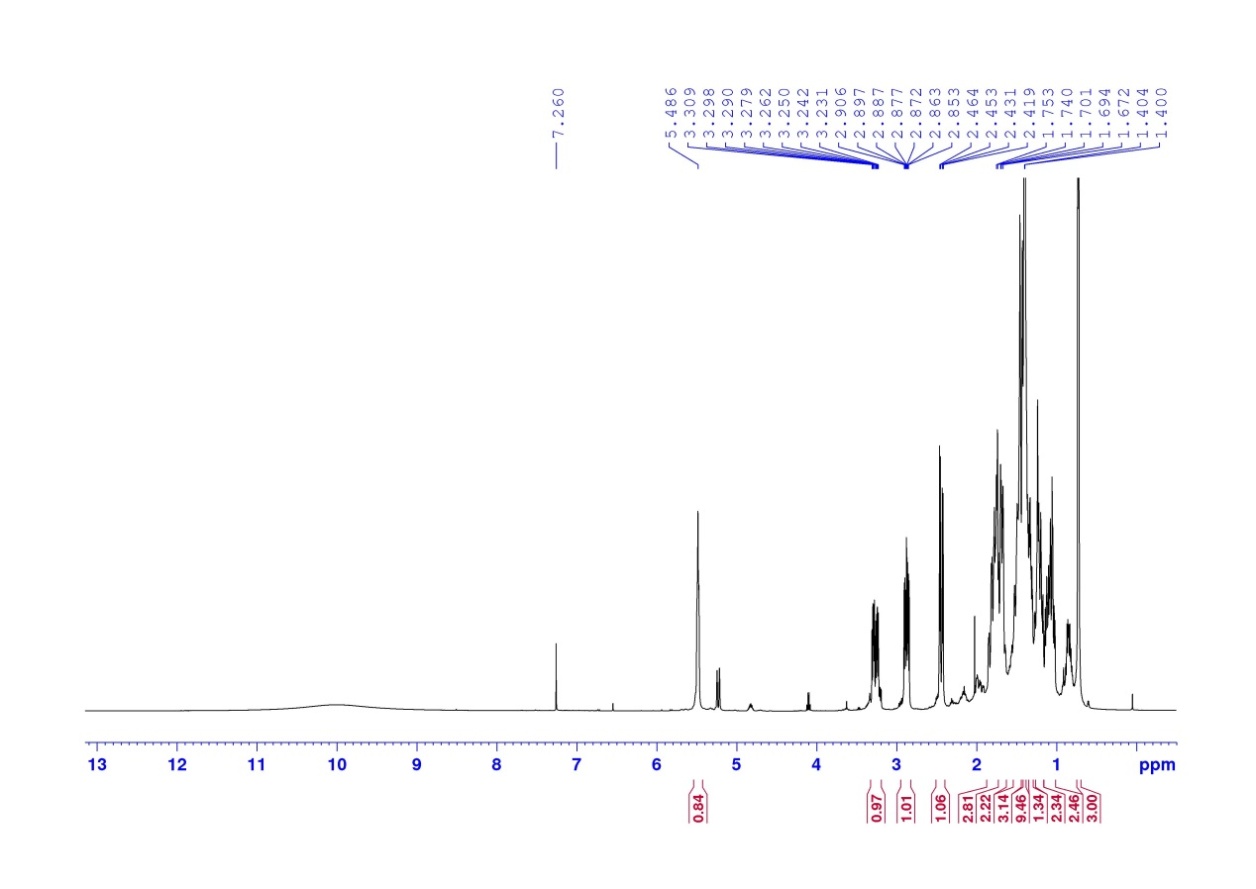


**^13^C NMR spectrum of compound *trans* 4a,b (500 MHz, CDCl_3_)**


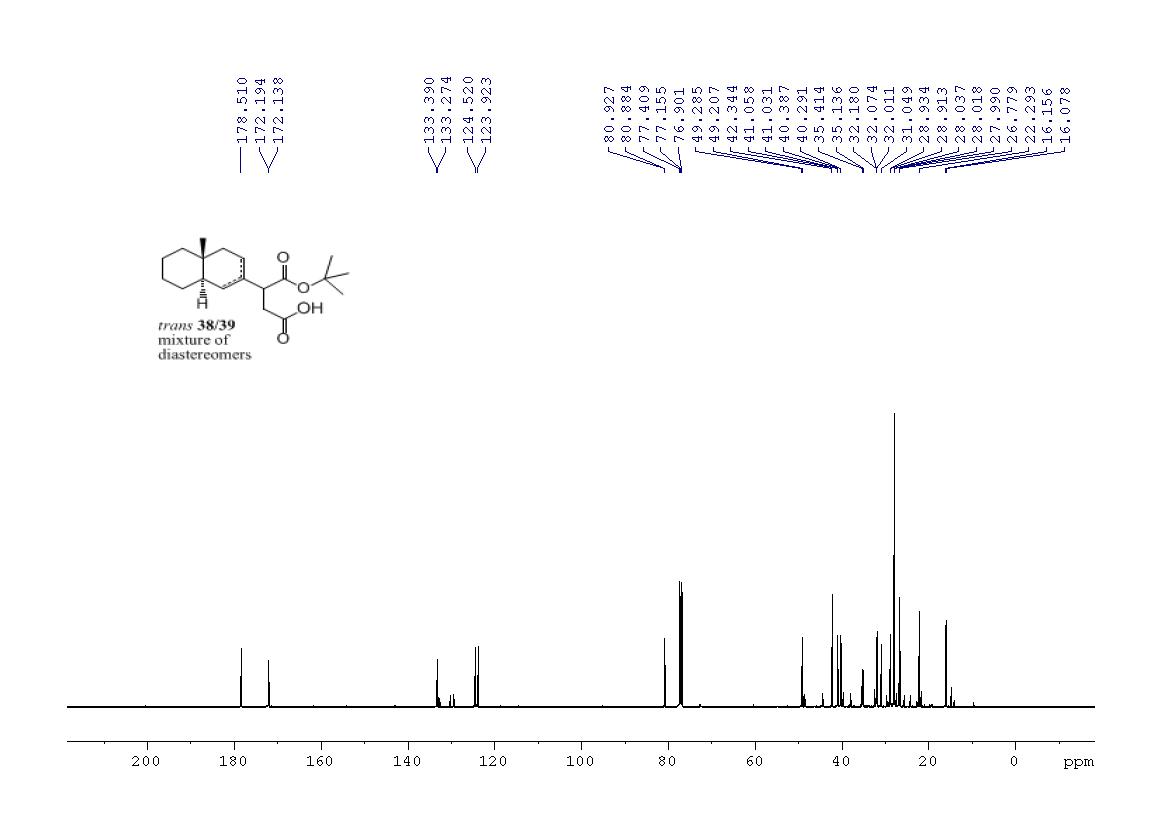


**^1^H NMR spectrum of compound *trans* 5a,b (500 MHz, CDCl_3_)**


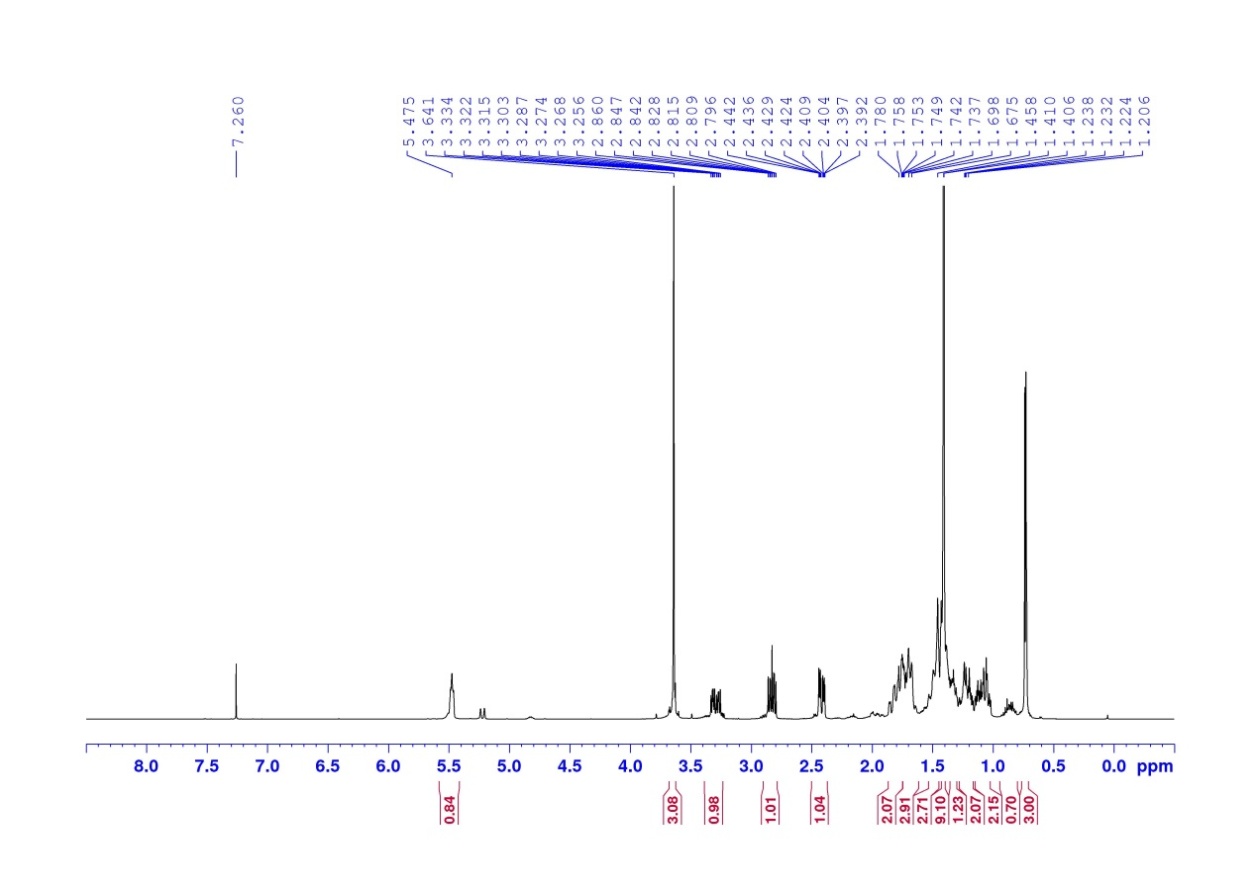
**^13^C NMR spectrum of compound *trans* 5a,b (500 MHz, CDCl_3_)**


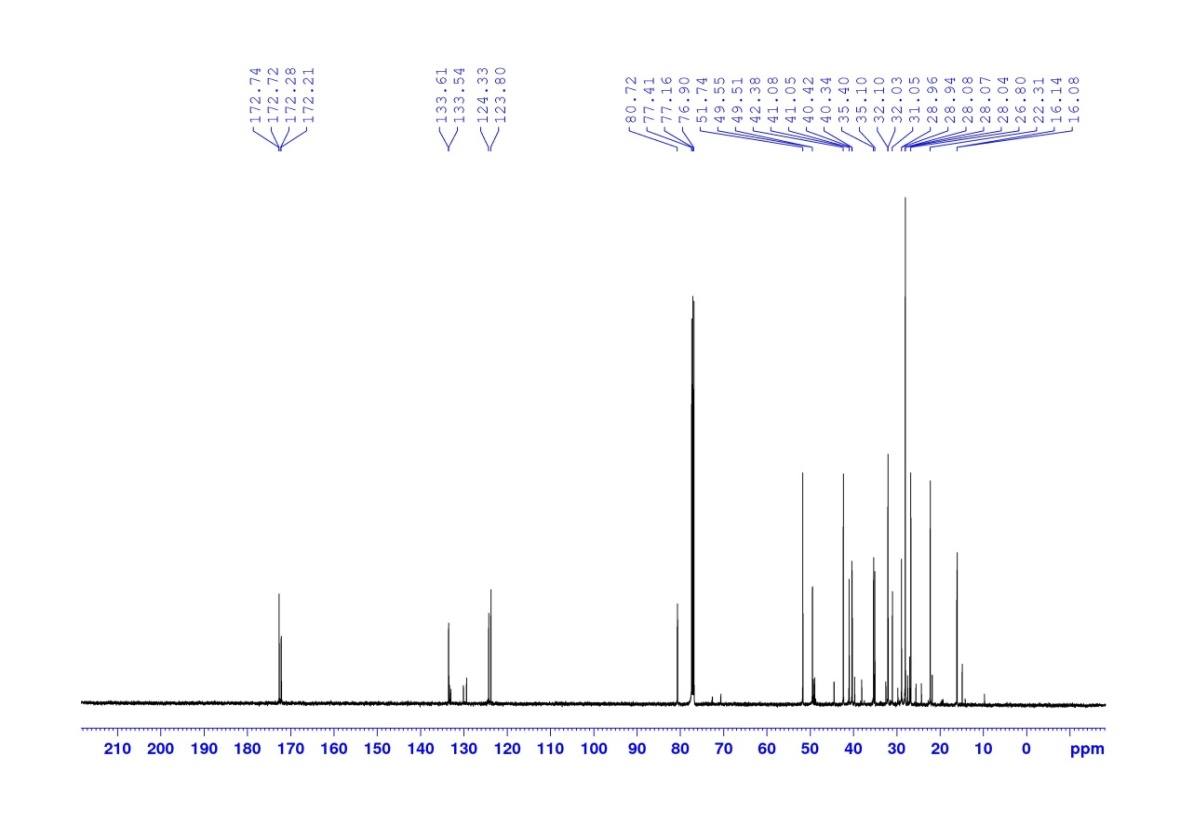


**^1^H NMR spectrum of compound *trans* 6 (500 MHz, CDCl_3_)**


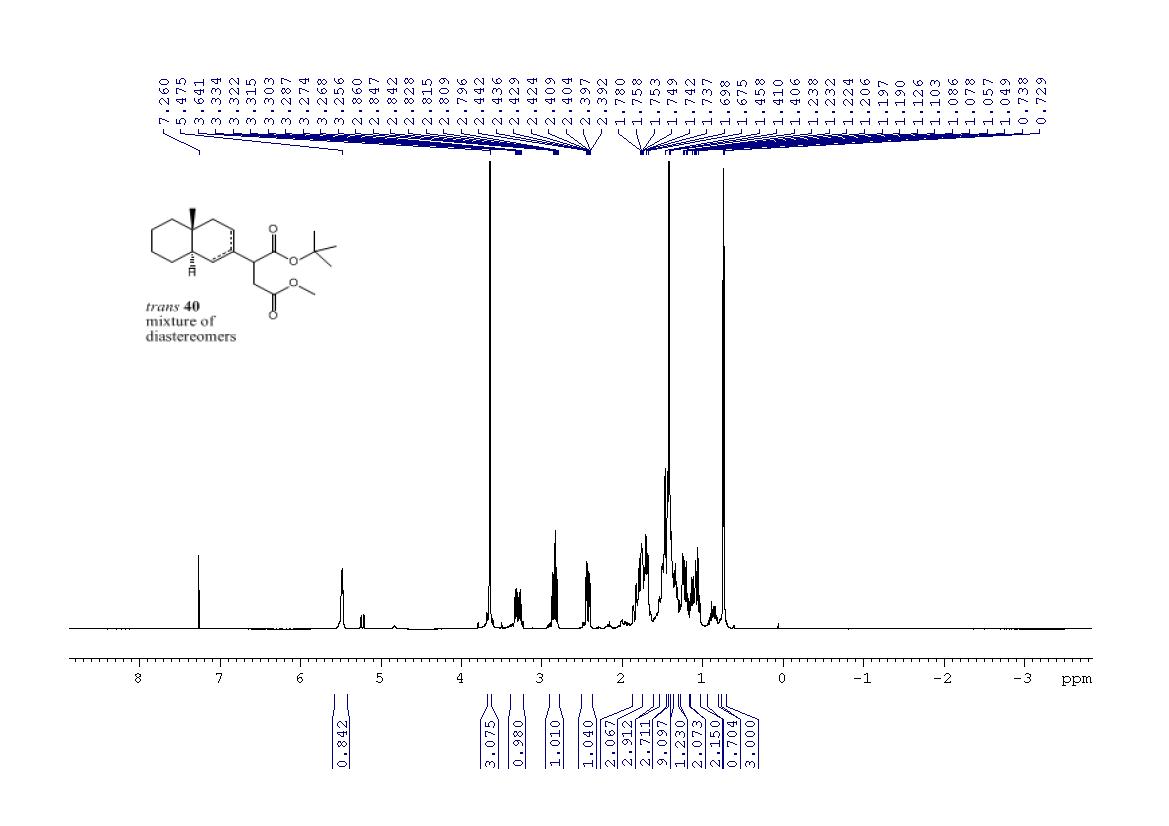


**^13^C NMR spectrum of compound *trans* 6 (500 MHz, CDCl_3_)**

+
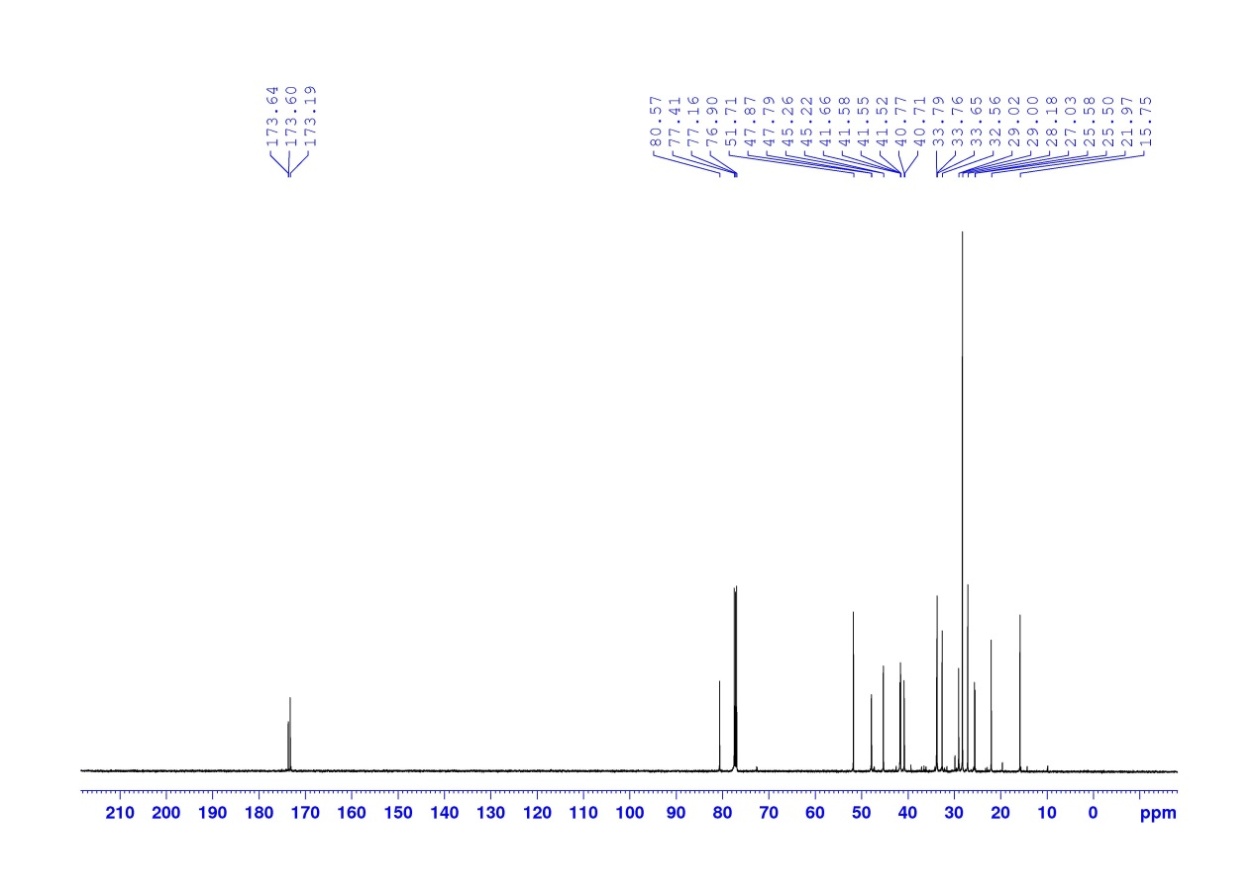


**DEPT135 spectrum of compound *trans* 6 (500 MHz, CDCl_3_)**


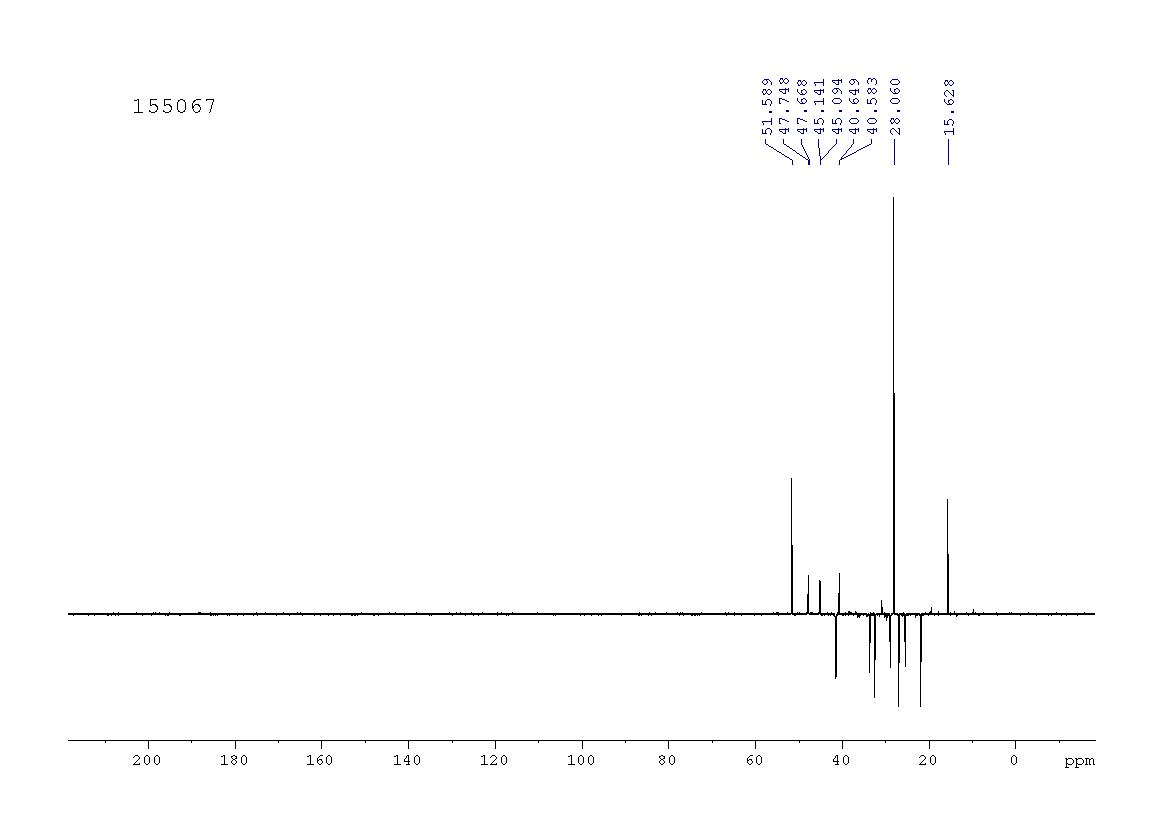


**COSY spectrum of compound *trans* 6 (500 MHz, CDCl_3_)**


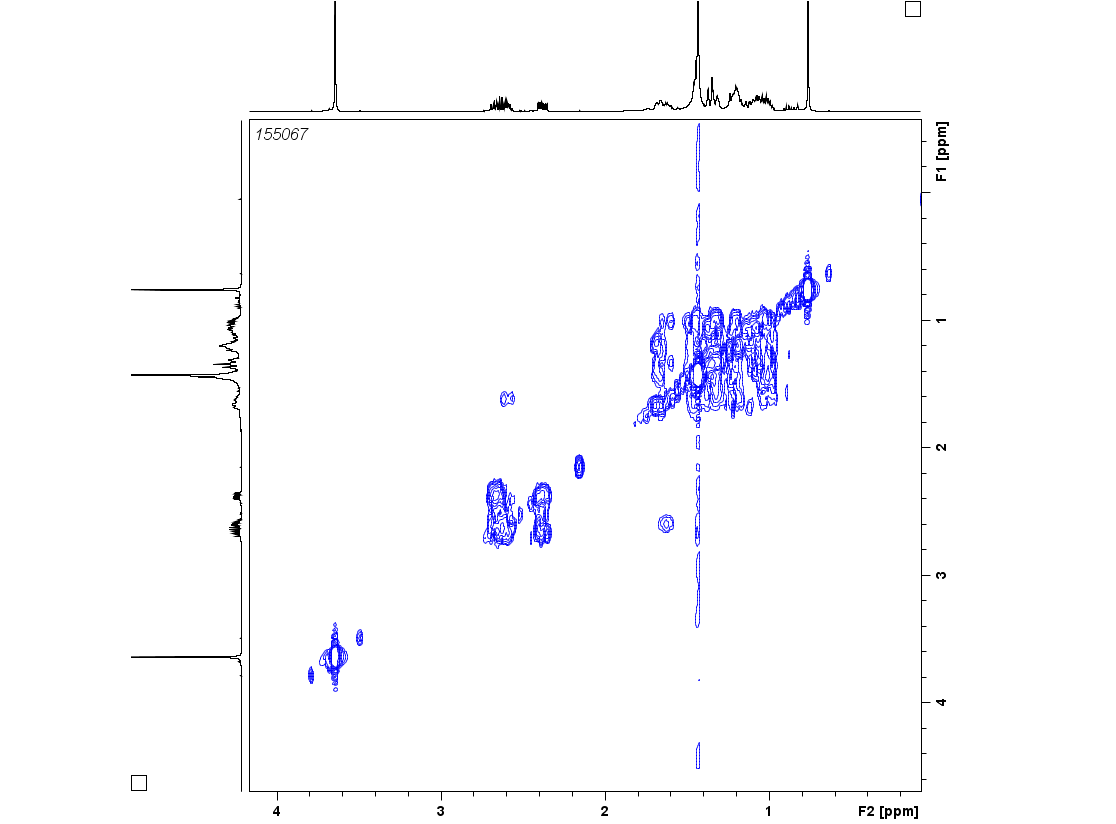


**HMBC spectrum of compound *trans* 6 (500 MHz, CDCl_3_)**


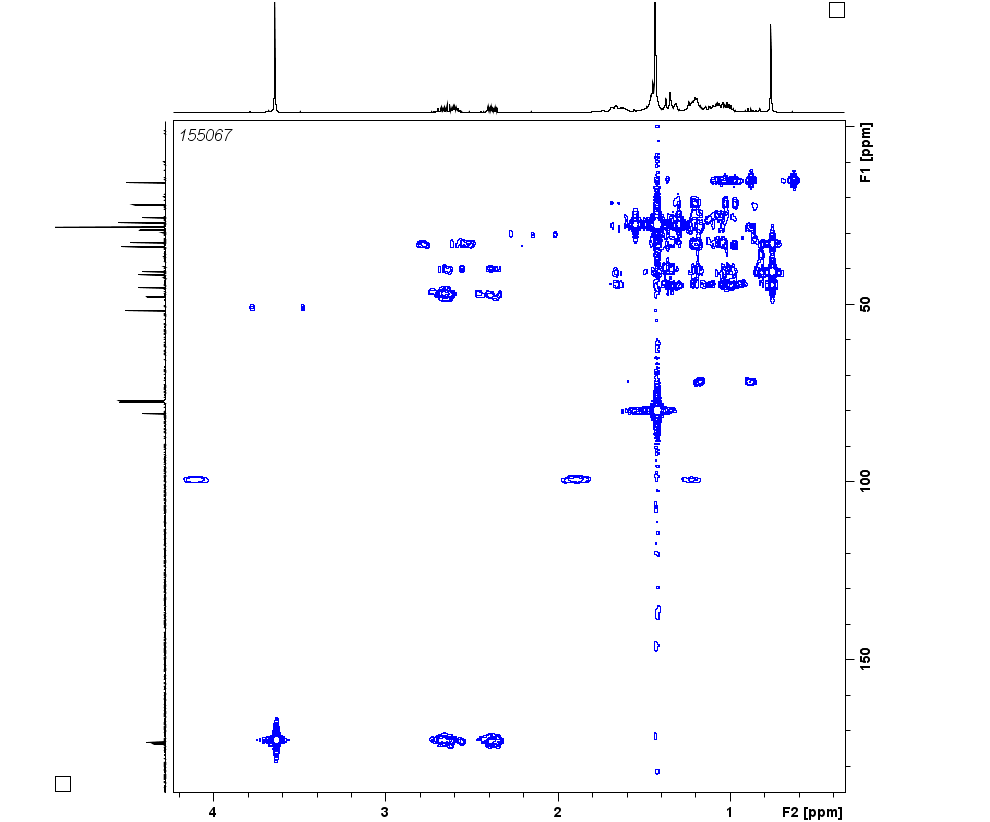


**HSQC spectrum of compound *trans* 6 (500 MHz, CDCl_3_)**


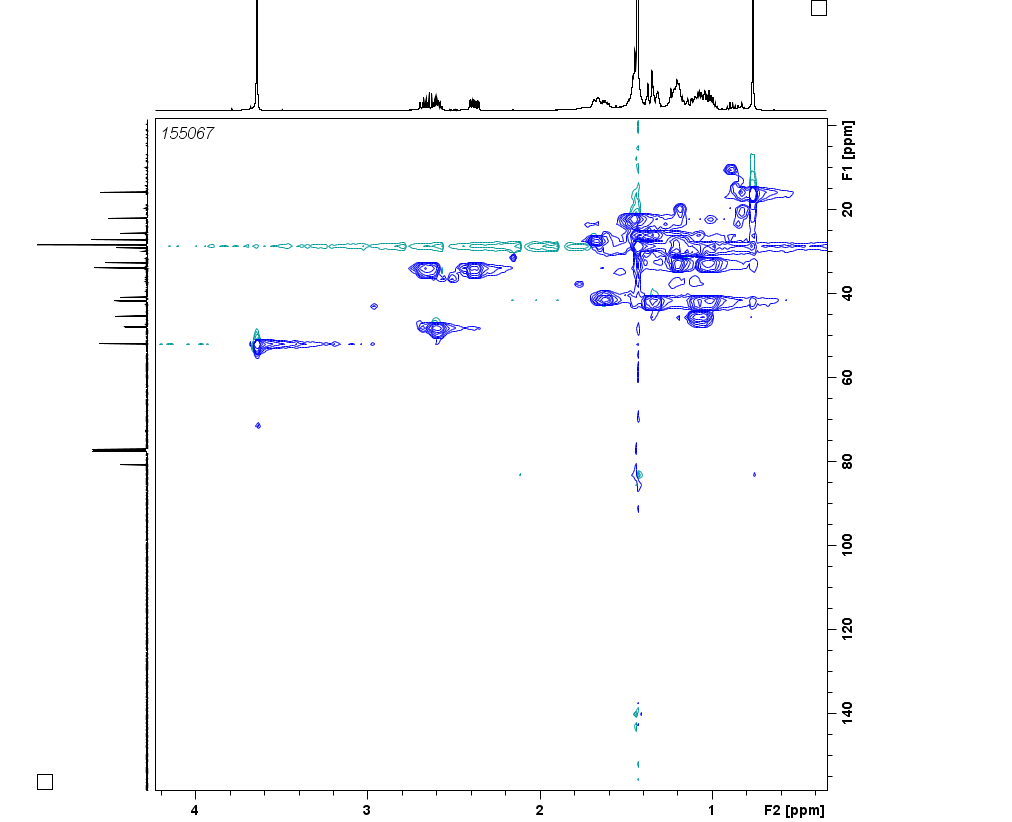


**NOESY spectrum of compound *trans* 6 (500 MHz, CDCl_3_)**


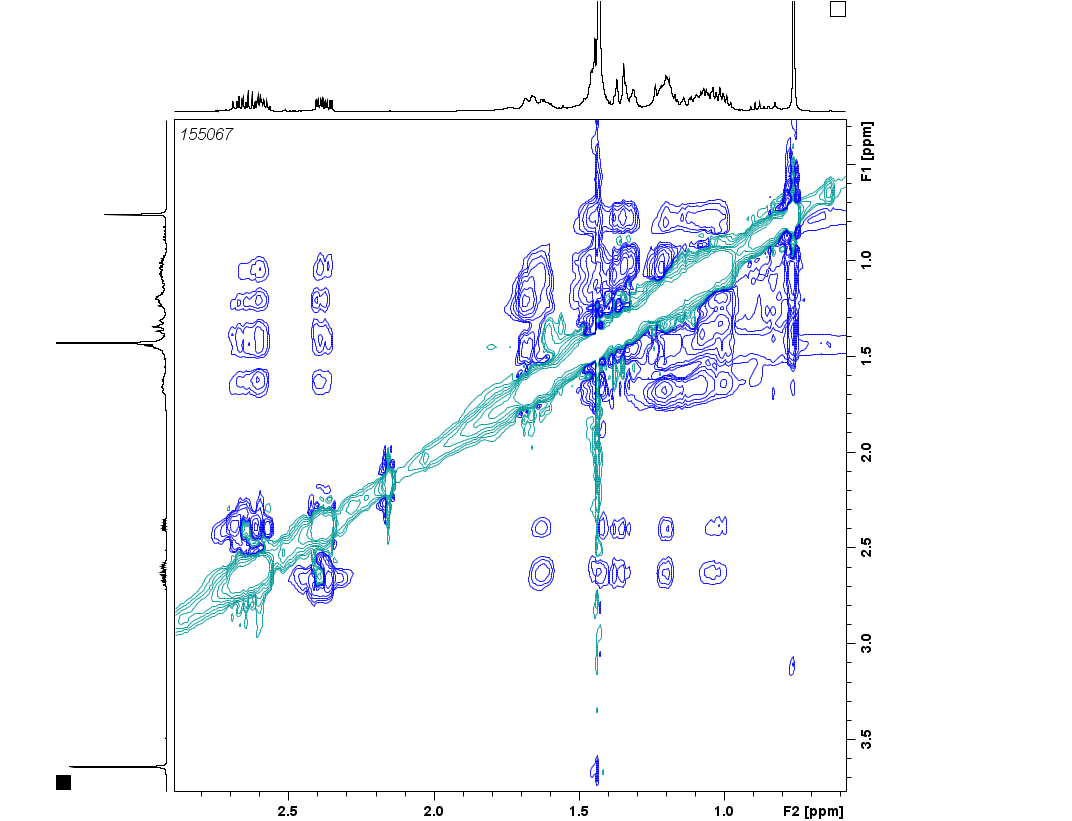


**^1^H NMR spectrum of compound *trans* 7 (500 MHz, CDCl_3_)**


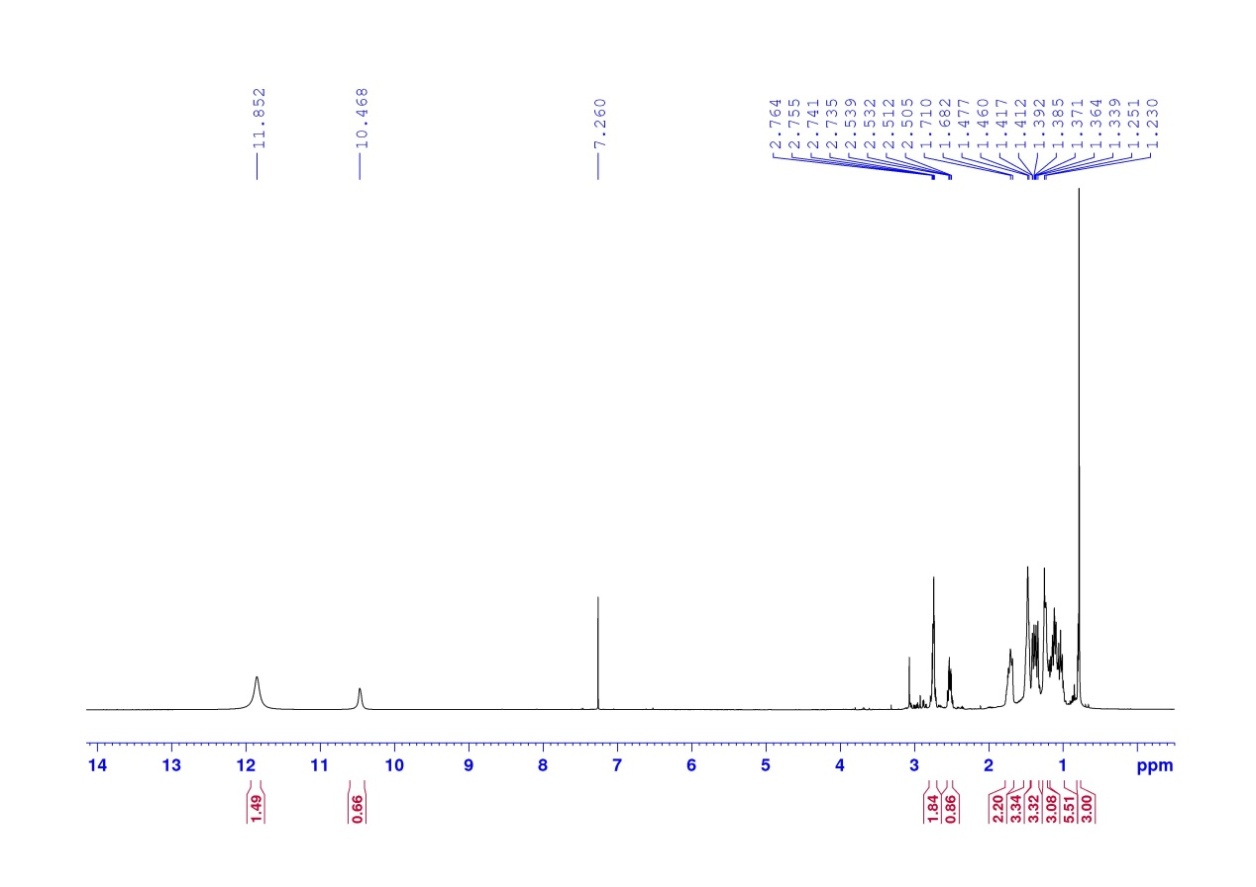


**^13^C NMR spectrum of compound *trans* 7 (500 MHz, CDCl_3_)**


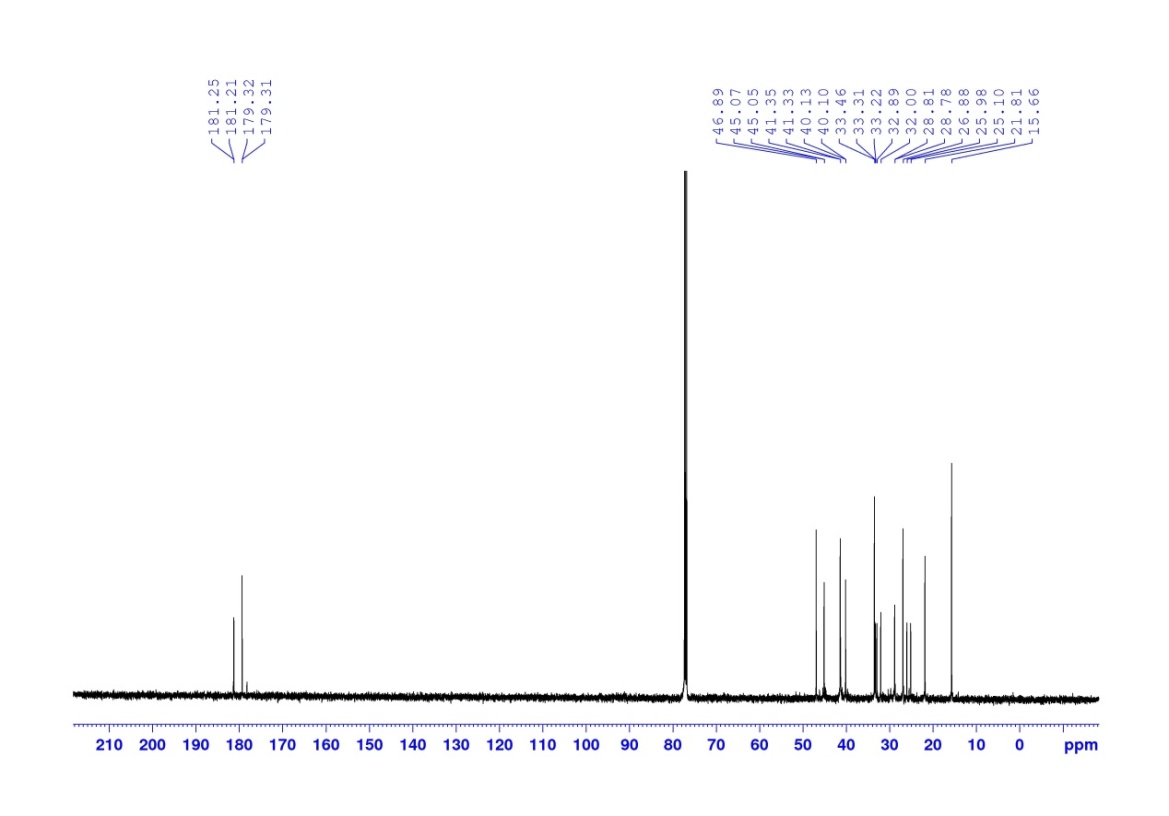


**^1^H NMR spectrum of compound (4aR,8aR)-2 (500 MHz, CDCl_3_)**


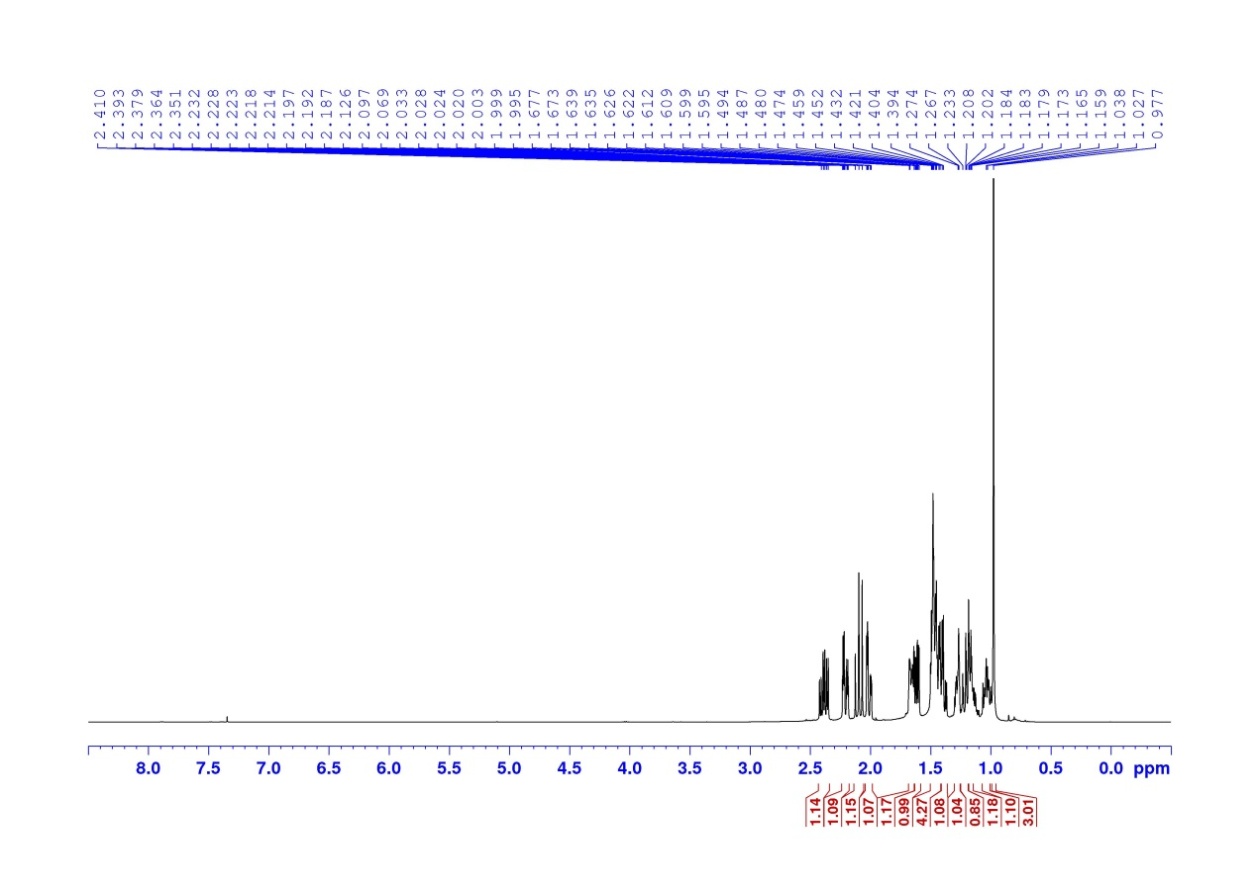
**^113^C NMR spectrum of compound (4aR, 8aR)-2 (500 MHz, CDCl_3_)**


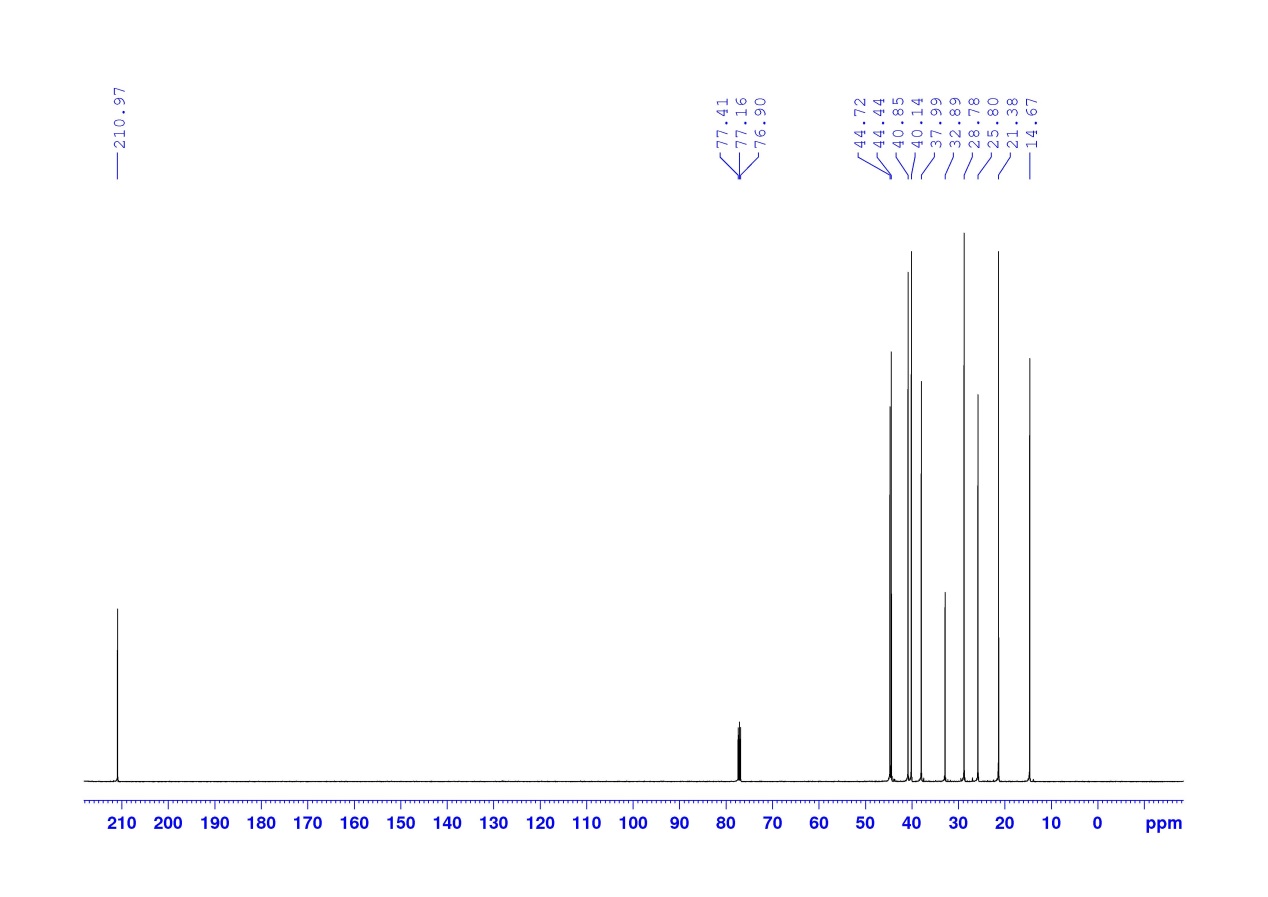


**^1^H NMR spectrum of compound (4aR, 8aR)-4a,b (500 MHz, CDCl_3_)**


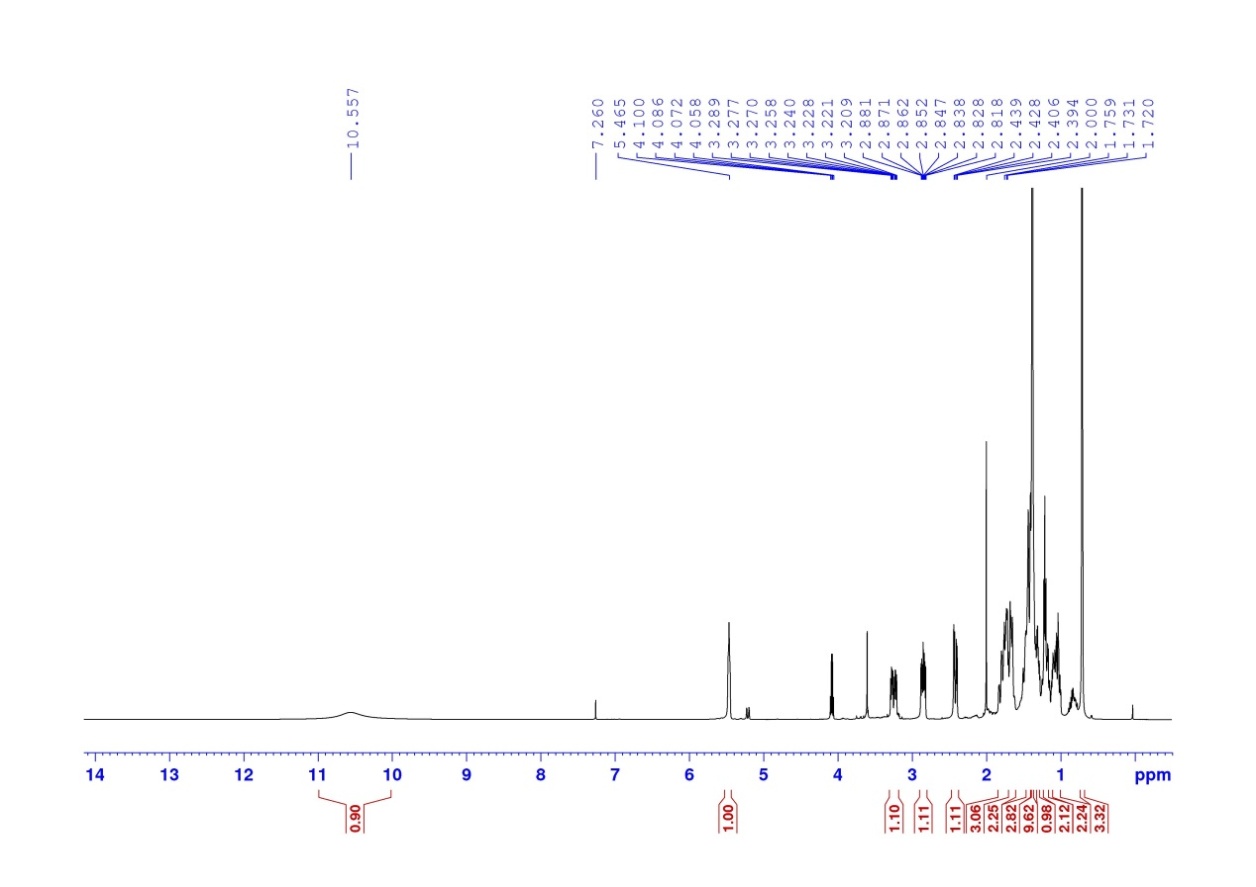
**^113^C NMR spectrum of compound (4aR, 8aR)-4a,b (500 MHz, CDCl_3_)**


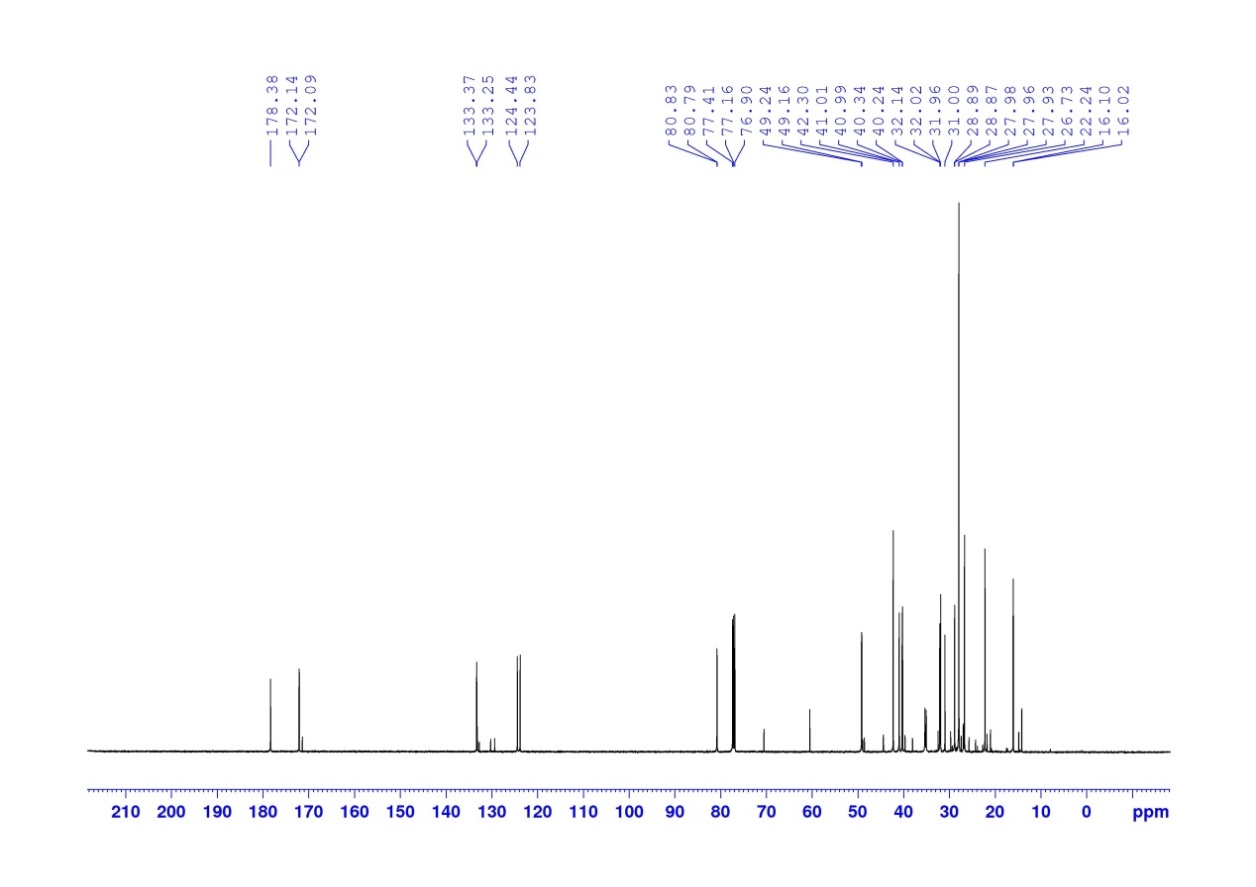


**^1^H NMR spectrum of compound (4aR, 8aR)-5a,b (500 MHz, CDCl_3_)**


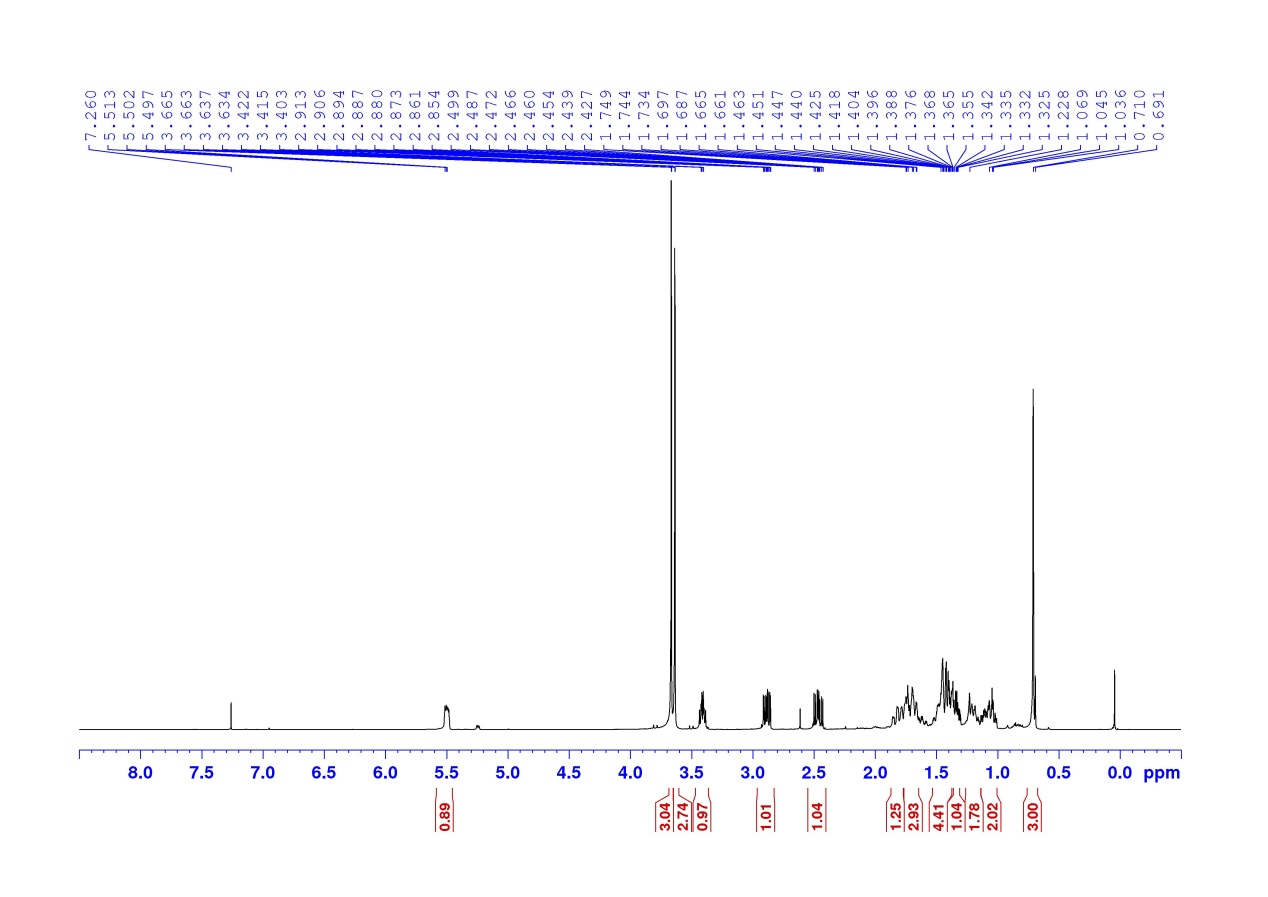


**^1^H NMR spectrum of compound (2R,4aR, 8aR)-6 (500 MHz, CDCl_3_)**


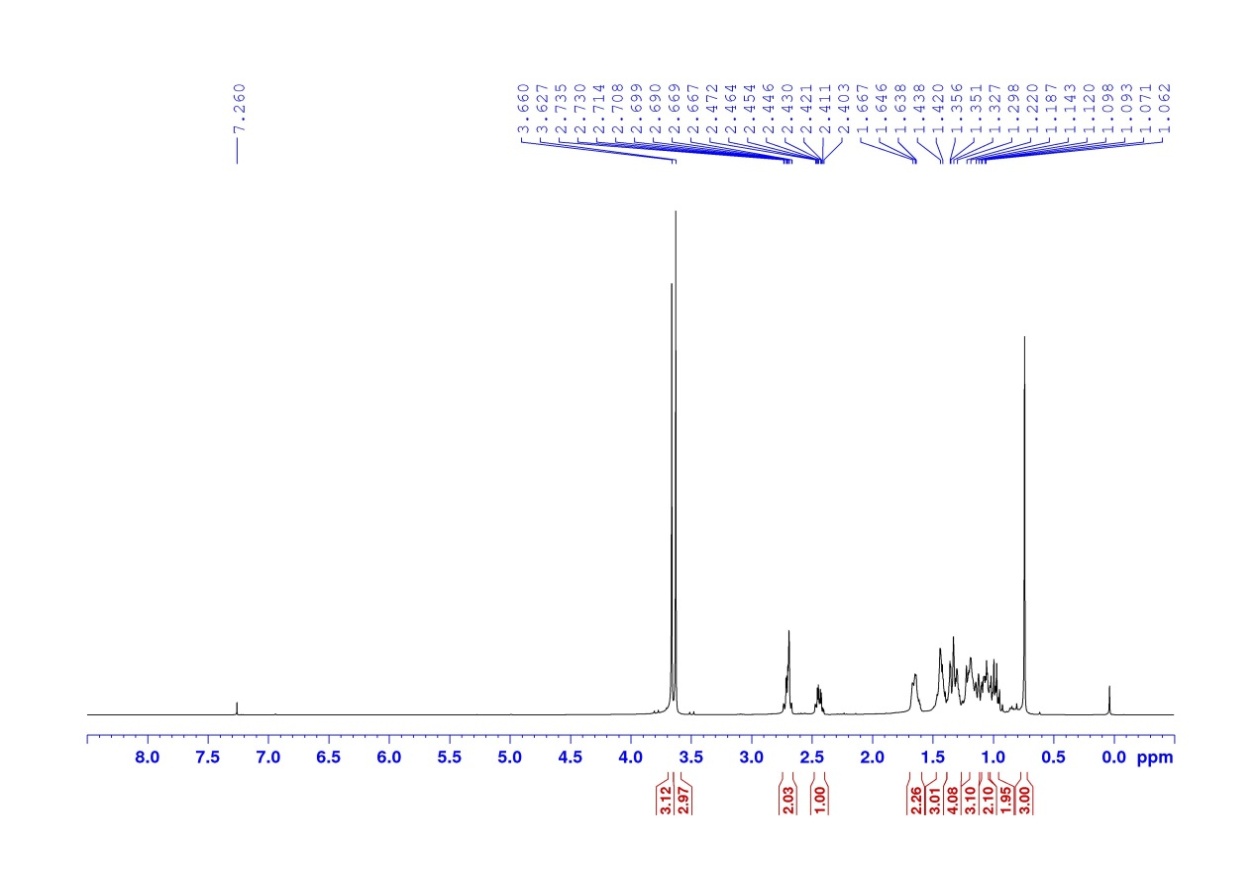
**^113^C NMR spectrum of compound (2R,4aR, 8aR)-6 (500 MHz, CDCl_3_)**


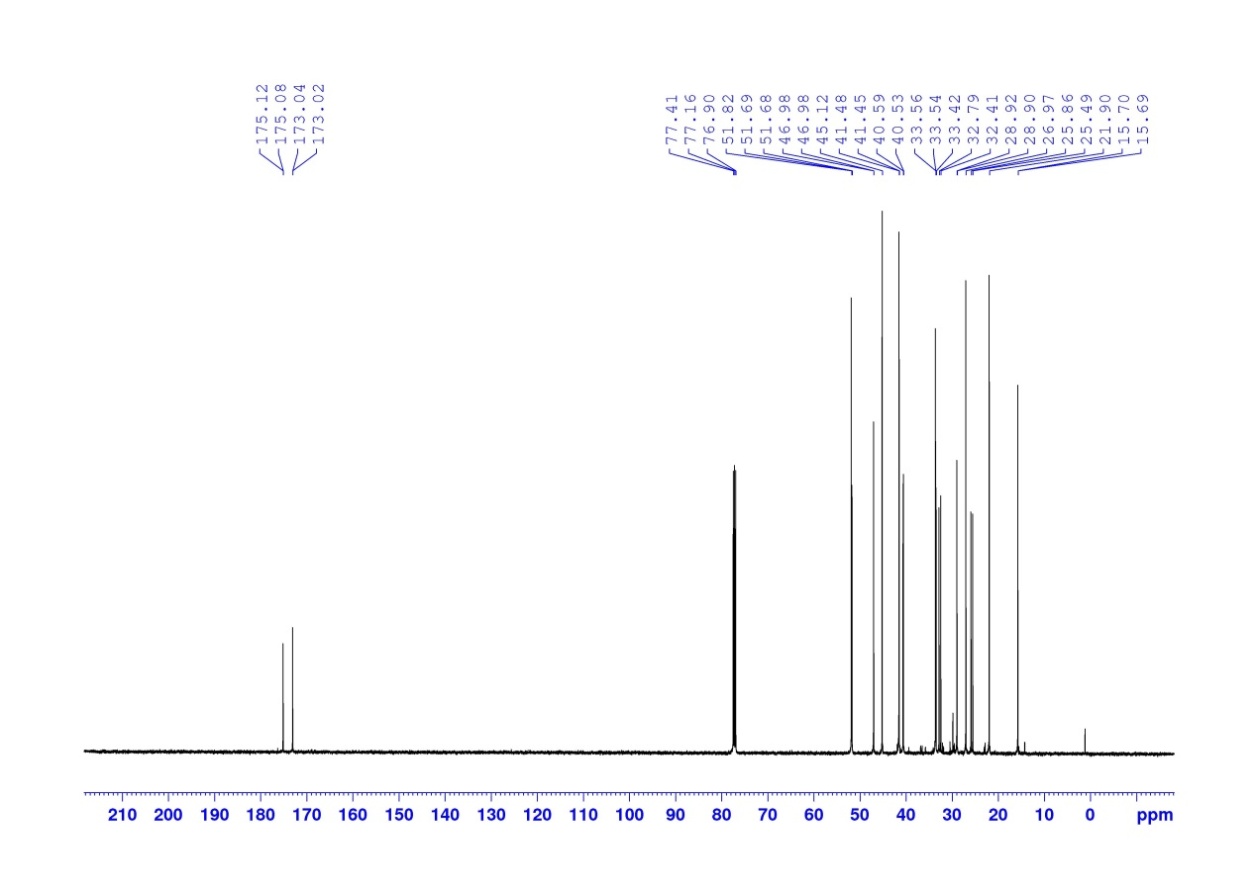


**DEPT 135 spectrum of compound (2R,4aR, 8aR)-6 (500 MHz, CDCl_3_)**


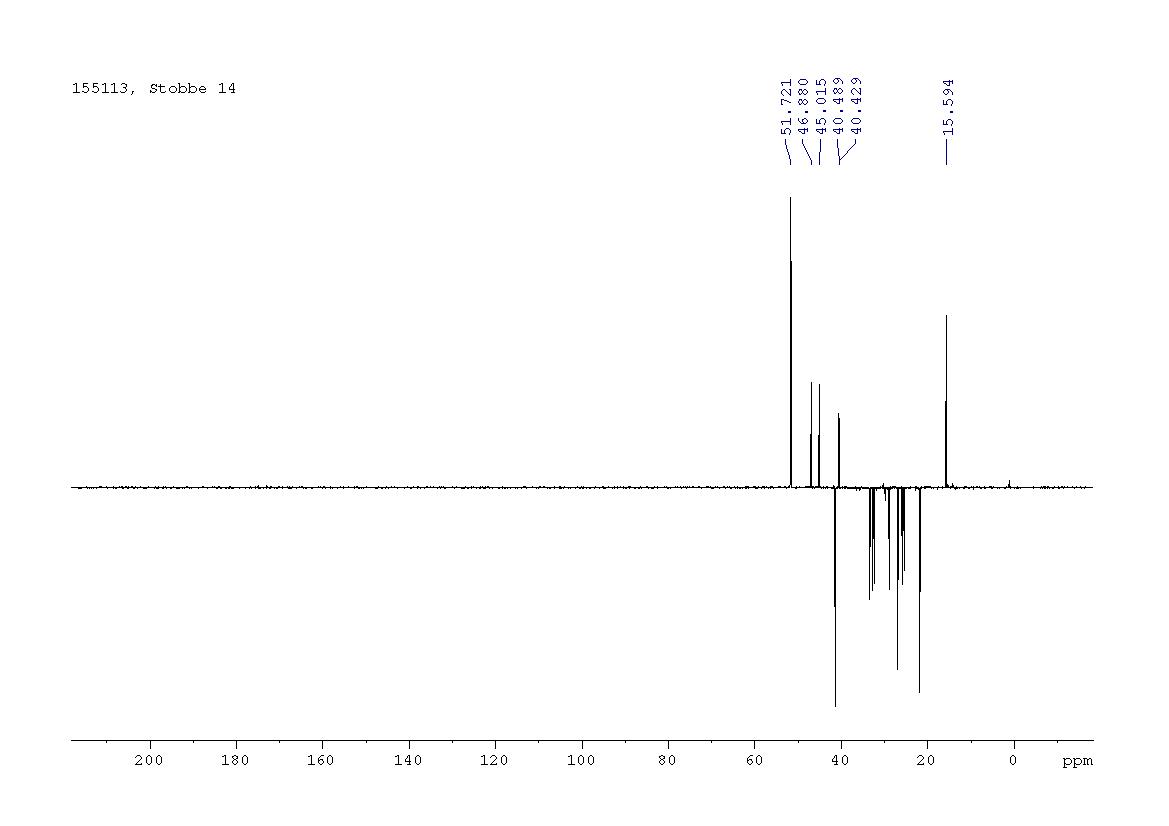


**COSY spectrum of compound (2R,4aR, 8aR)-6 (500 MHz, CDCl_3_)**


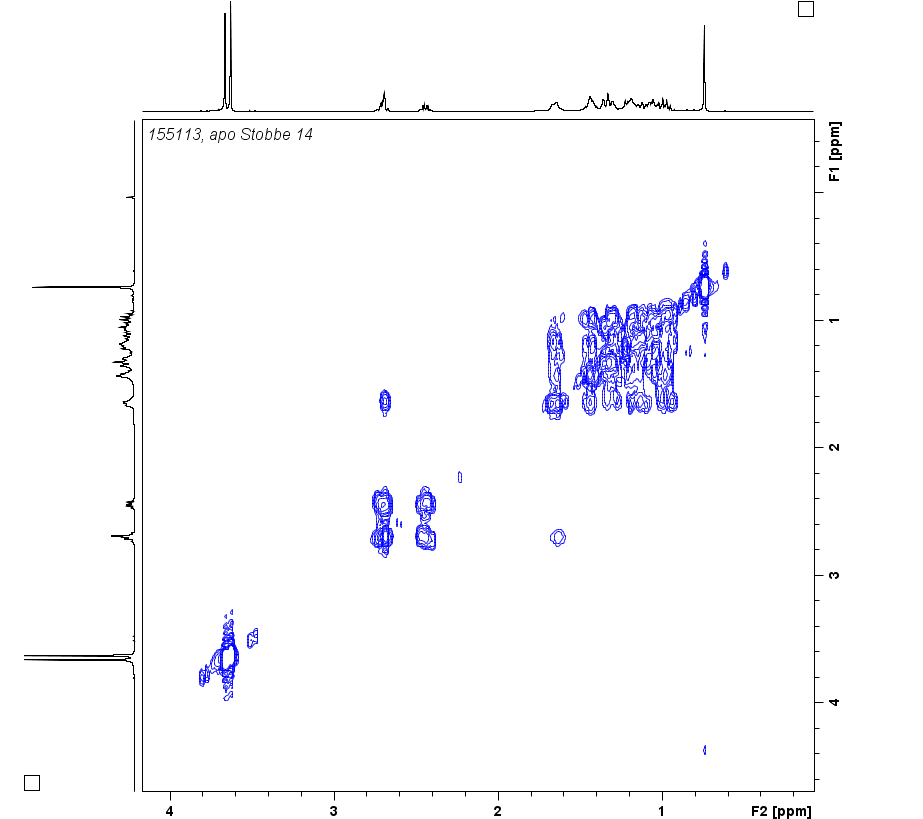


**HMBC spectrum of compound (2R,4aR, 8aR)-6 (500 MHz, CDCl_3_)**


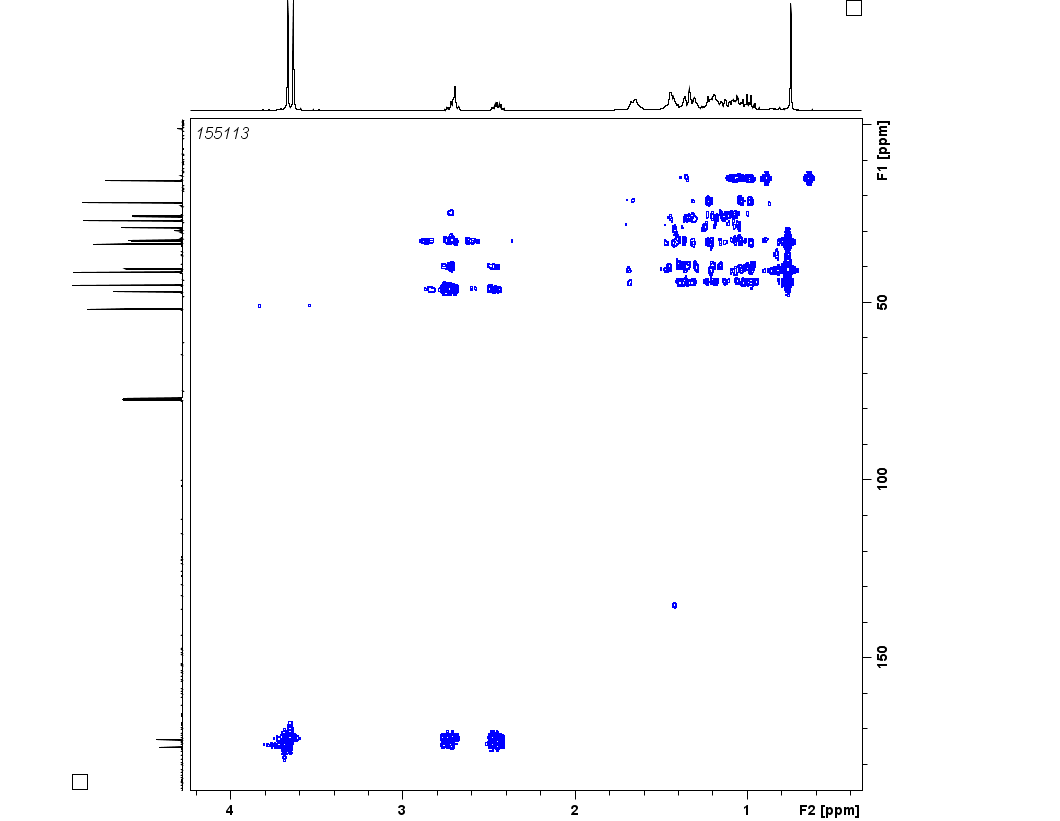


**HSQC spectrum of compound (2R,4aR, 8aR)-6 (500 MHz, CDCl_3_)**


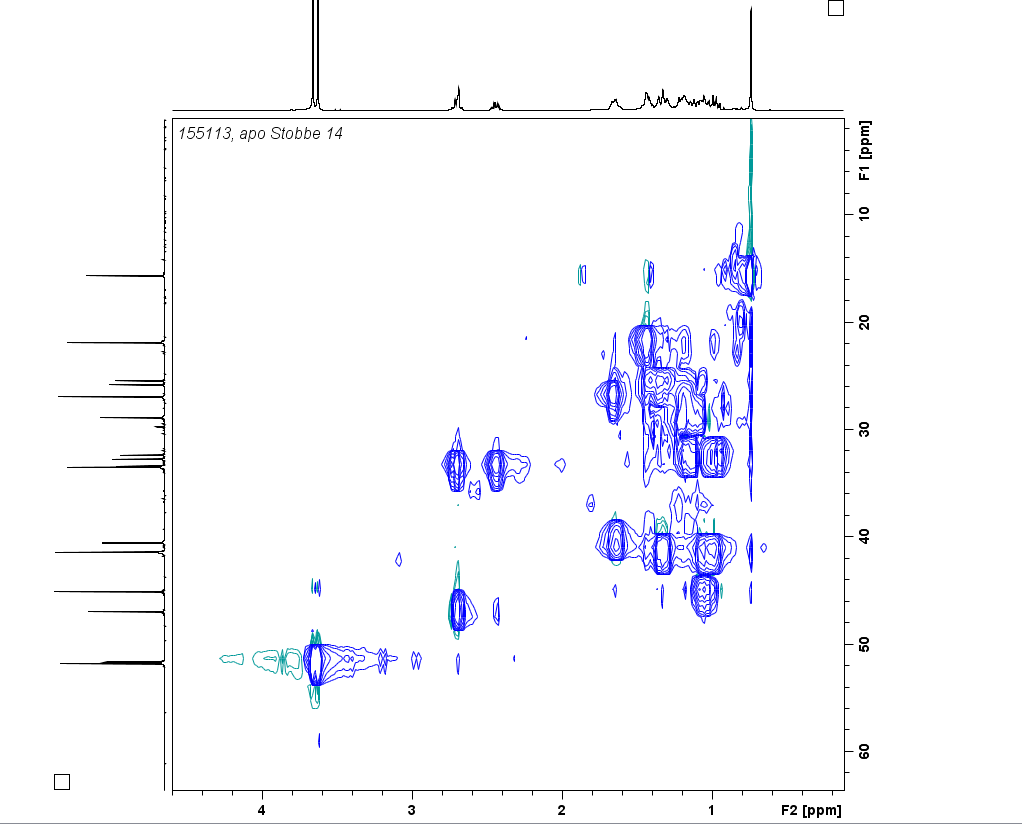


**NOESY spectrum of compound (2R,4aR, 8aR)-6 (500 MHz, CDCl_3_)**


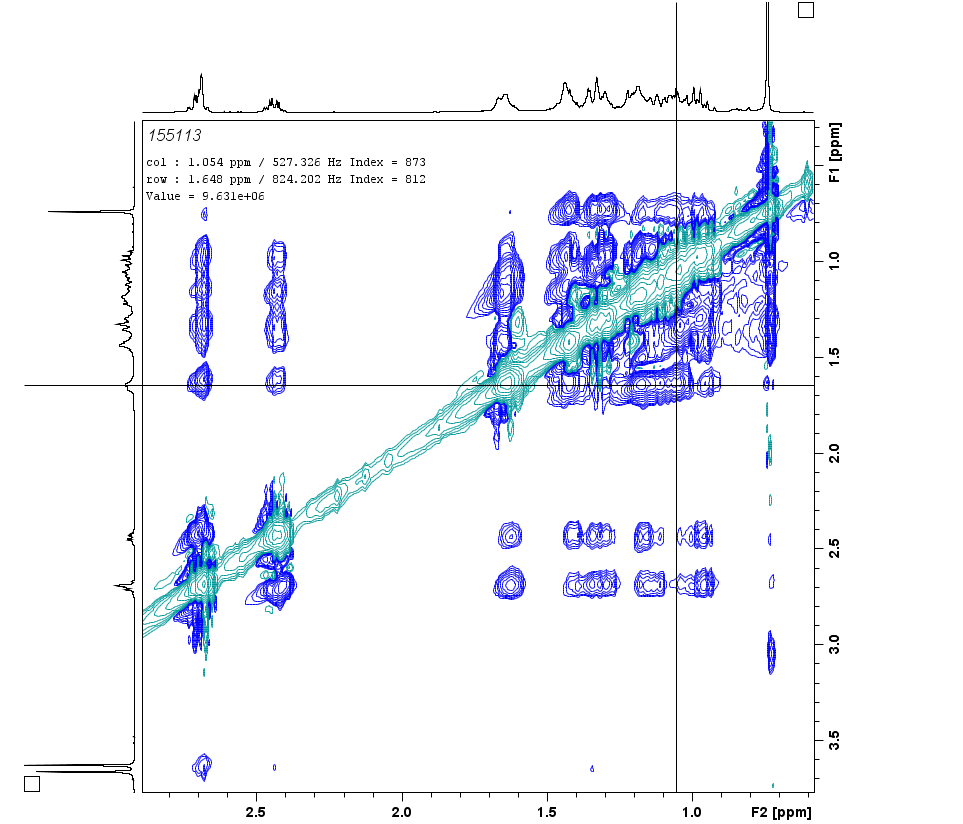


**^1^H NMR spectrum of compound (2R,4aR, 8aR)-7 (500 MHz, CDCl_3_)**


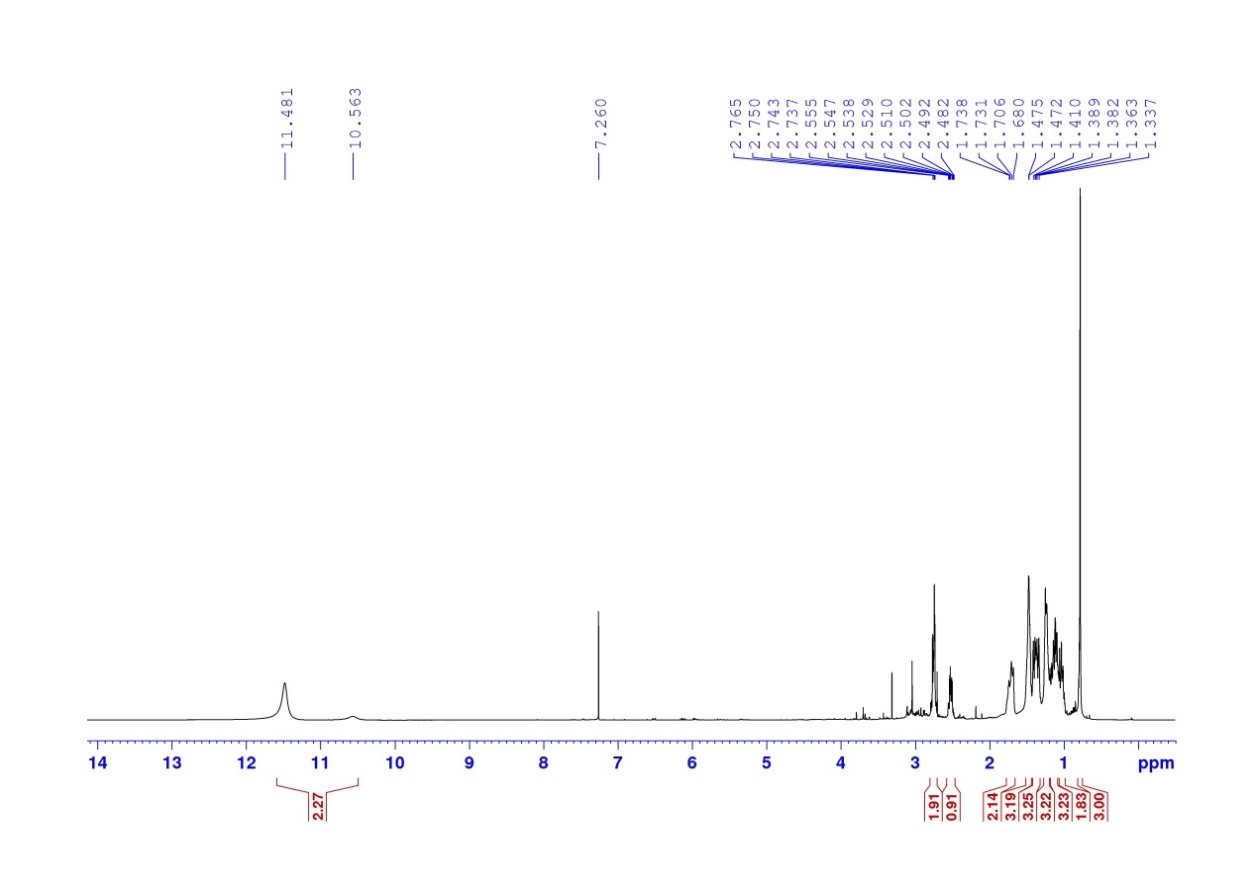
**^113^C NMR spectrum of compound (2R,4aR, 8aR)-7 (500 MHz, CDCl_3_)**


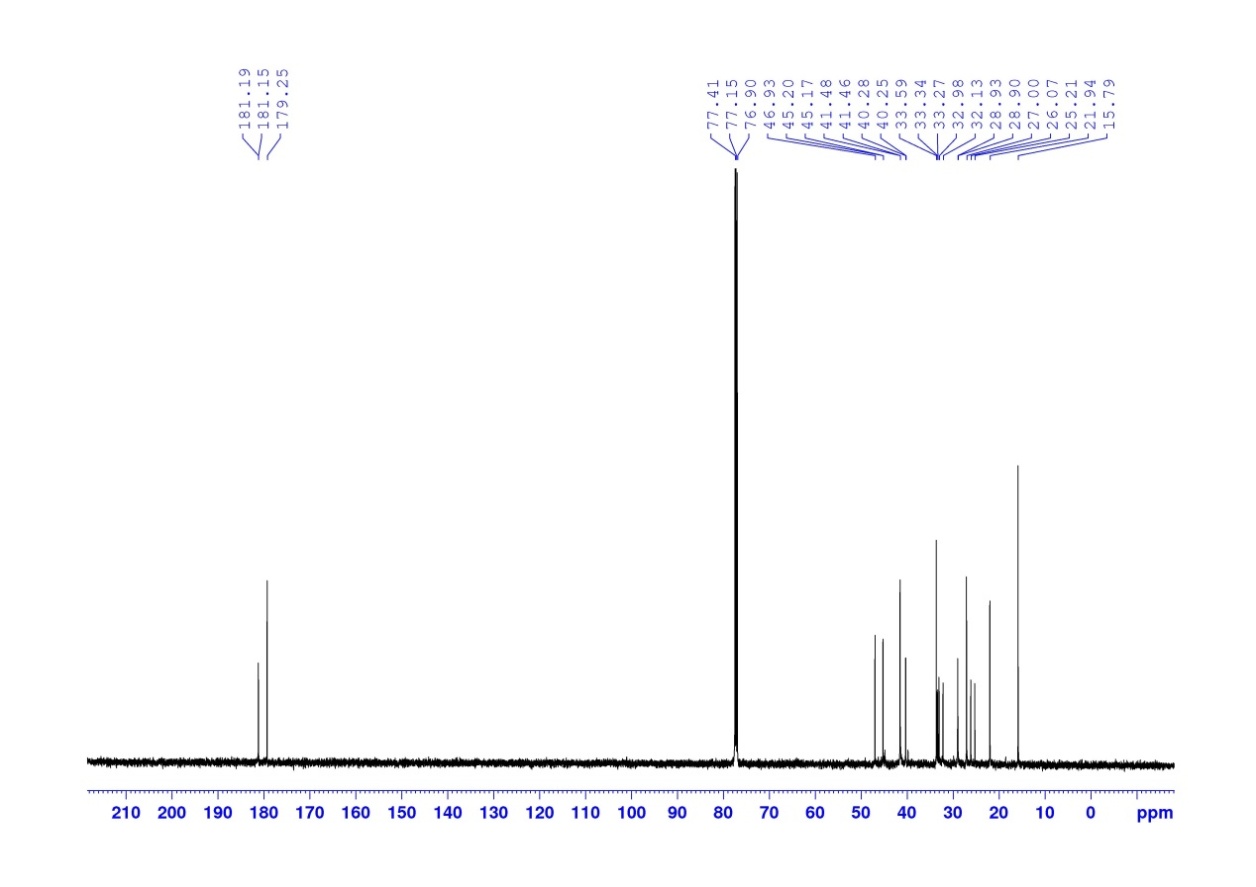


**DEPT135 spectrum of compound (2R,4aR, 8aR)-7 (500 MHz, CDCl_3_)**


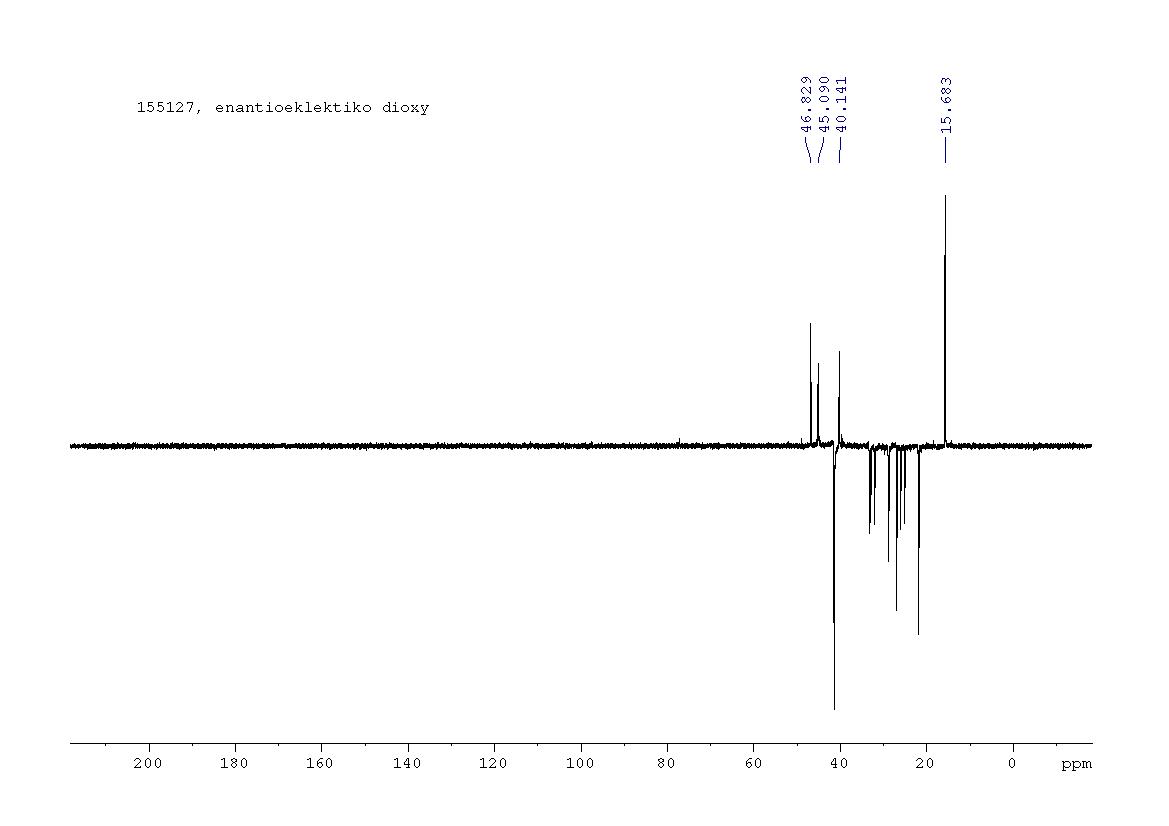
**COSY spectrum of compound (2R,4aR, 8aR)-7 (500 MHz, CDCl_3_)**


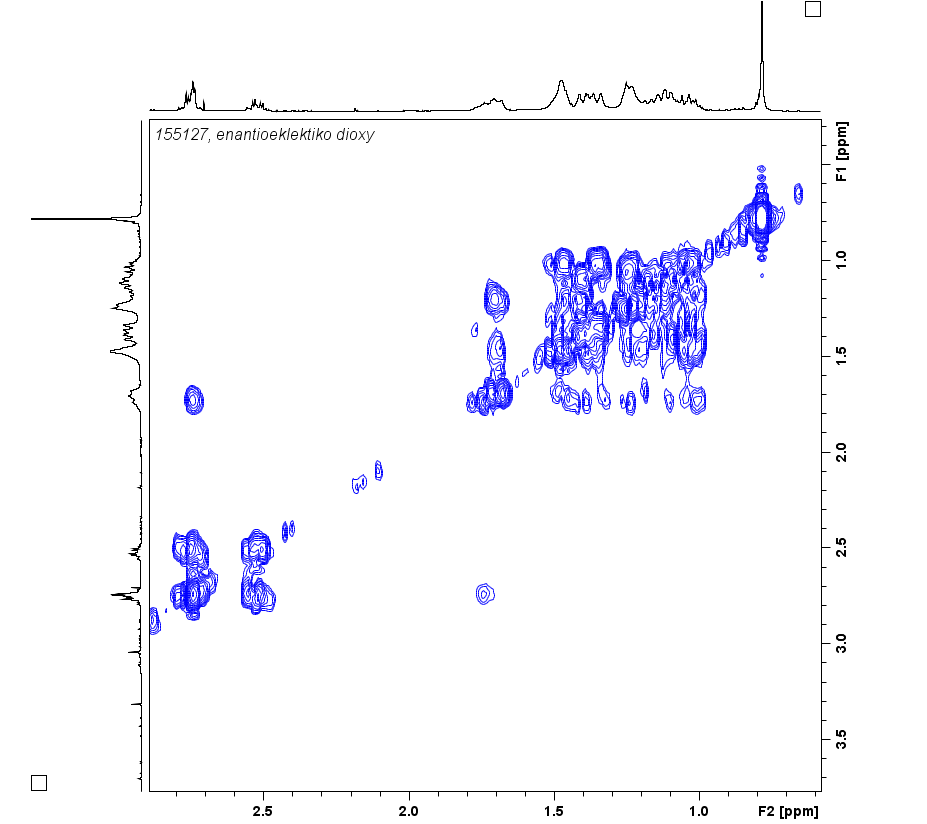


**HMBC spectrum of compound (2R,4aR, 8aR)-7 (500 MHz, CDCl_3_)**


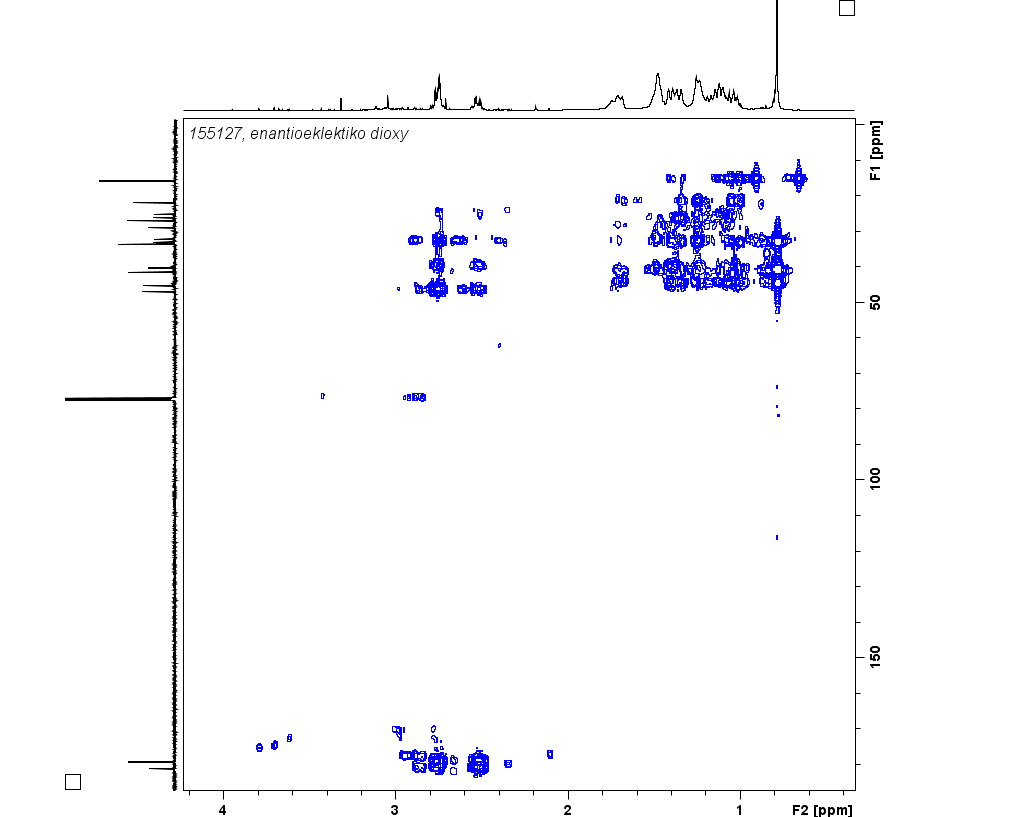


**HSQC spectrum of compound (2R,4aR, 8aR)-7 (500 MHz, CDCl_3_)**


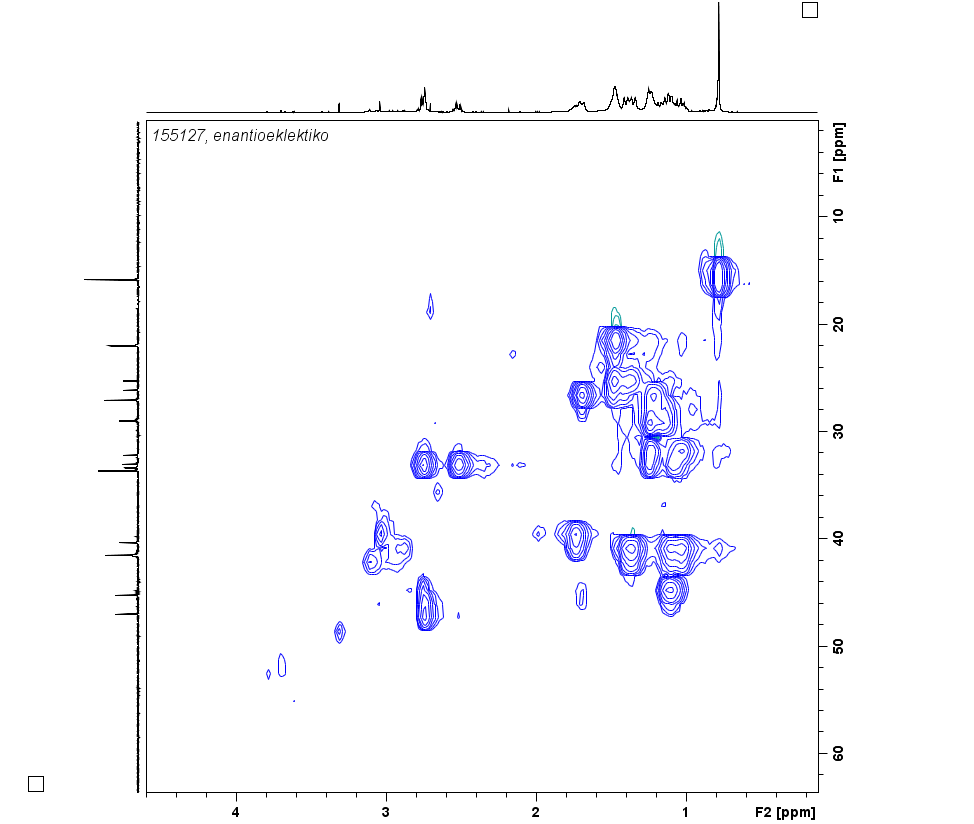


**NOESY spectrum of compound (2R,4aR, 8aR)-7 (500 MHz, CDCl_3_)**


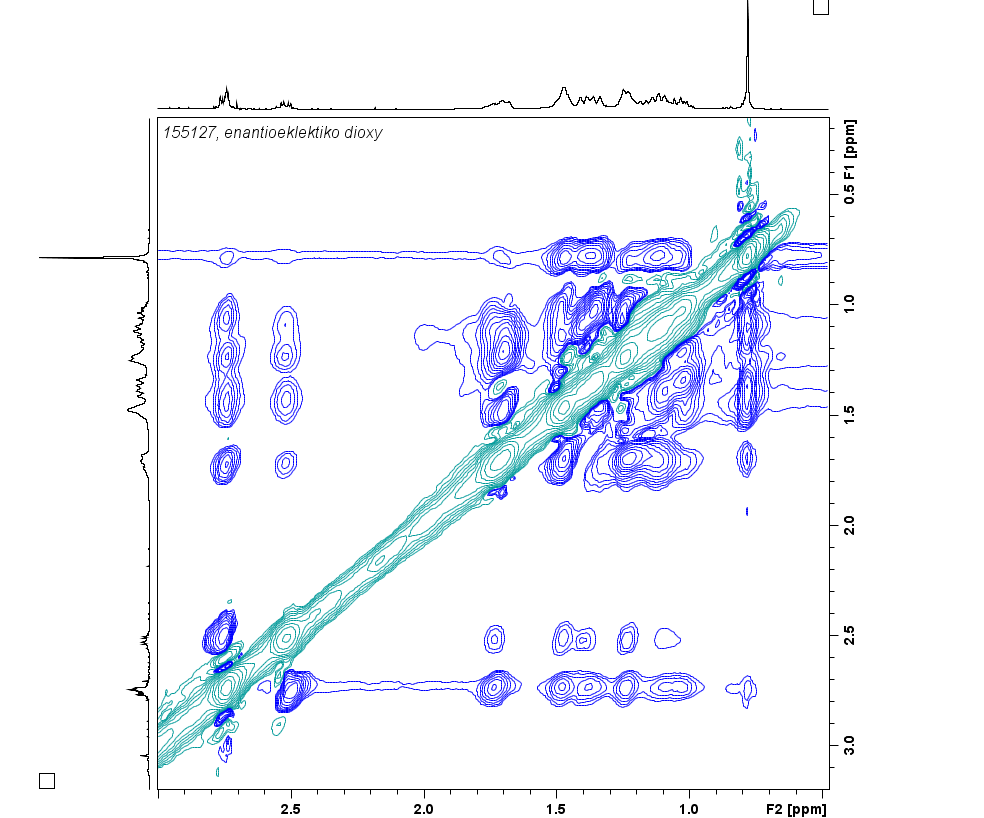


A

B


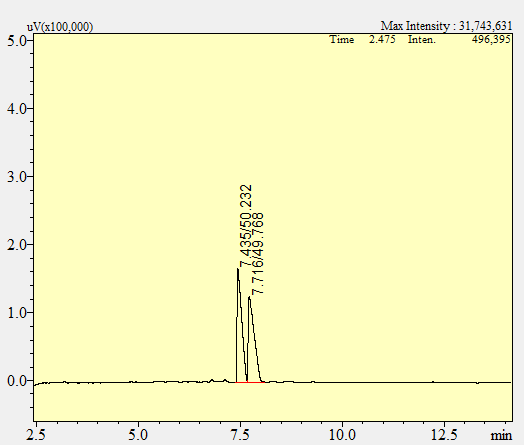


C


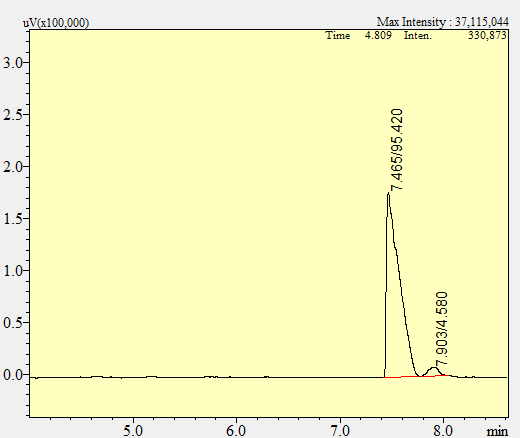


D


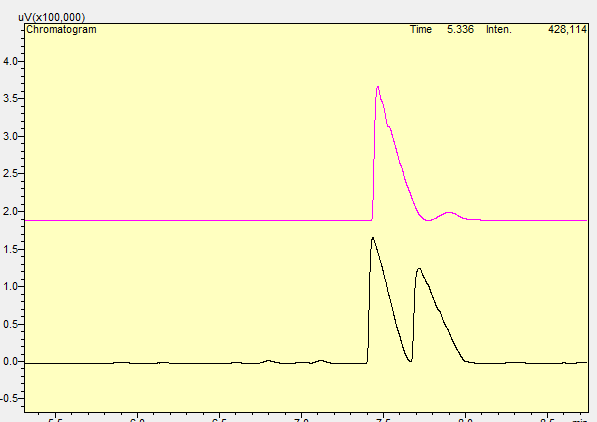


A: GC analysis of ***trans*-2** on a non-chiral column, conditions described in the “General Procedures” section, indicating the presence of the two major diastereomers in a ratio of 96.4/2.04

B-D: GC analysis on chiral column, conditions described in the “General Procedures” section.

B: chromatogram of ***trans*-2**, indicating the presence of the two enantiomers of the major diastereomer in a ratio of 50.2/49.8

C: chromatogram of **(4a*R*,8a*R)*-2** indicating the presence of the enantiomers in a ratio of 95.4/4.6

D: overlap of the two chromatograms

F

G

F: GC analysis of ***trans*-6** on a non-chiral column, conditions described in the “General Procedures” section, indicating the presence of the two major diastereomers in a ratio of 83.9/16.1

G: HPLC analysis on chiral column, conditions described in the “General Procedures” section. Chromatogram of ***trans*-6**, indicating the presence of the two enantiomeric pairs

H

I

H: GC analysis of **(2*R*,4α*R*,8α*R*)-6** on a non-chiral column, conditions described in the “General Procedures” section, indicating the presence of two diastereomers in a ratio of 95.96/4.04

I: HPLC analysis on chiral column, conditions described in the “General Procedures” section. Chromatogram of **(2*R*,4α*R*,8α*R*)-6**, indicating the presence of one enantiomer with retention time of 22.15 min, the same as the *first* of the two enantiomers of the major diastereomers.

REFERENCES

1. Voutsadaki S, Tsikalas GK, E Klontzas E, Froudakis GE, Pergantis SA, Demadis KD, Katerinopoulos HE. 2012 A cyclam-type “turn on” fluorescent sensor selective for mercury ions in aqueous media. *RSC Adv*. **2**, 12679-12682.

Revial G, Pfau M. 1992 (R)-(−)-10-Methyl-1(9)-octal-2-one [2(3H)-Naphthalenone,4,4a,5,6,7,8-hexahydro-4a-methyl, (R)-] *Org. Synth.* **70**, 35.
